# Glial loss of the metallo β-lactamase domain containing protein, SWIP-10, induces age- and glutamate-signaling dependent, dopamine neuron degeneration

**DOI:** 10.1371/journal.pgen.1007269

**Published:** 2018-03-28

**Authors:** Chelsea L. Gibson, Joseph T. Balbona, Ashlin Niedzwiecki, Peter Rodriguez, Ken C. Q. Nguyen, David H. Hall, Randy D. Blakely

**Affiliations:** 1 Department of Pharmacology, Vanderbilt University, Nashville, TN, United States of America; 2 Department of Biomedical Science, Charles E. Schmidt College of Medicine, Florida Atlantic University, Boca Raton, FL, United States of America; 3 Department of Neuroscience, Albert Einstein College of Medicine, Bronx, NY, United States of America; 4 Department of Psychiatry, Vanderbilt University, Nashville, TN, United States of America; 5 The Brain Institute, Florida Atlantic University, Jupiter, FL, United States of America; University of Miami School of Medicine, UNITED STATES

## Abstract

Across phylogeny, glutamate (Glu) signaling plays a critical role in regulating neural excitability, thus supporting many complex behaviors. Perturbed synaptic and extrasynaptic Glu homeostasis in the human brain has been implicated in multiple neuropsychiatric and neurodegenerative disorders including Parkinson’s disease, where theories suggest that excitotoxic insults may accelerate a naturally occurring process of dopamine (DA) neuron degeneration. In *C*. *elegans*, mutation of the glial expressed gene, *swip-10*, results in Glu-dependent DA neuron hyperexcitation that leads to elevated DA release, triggering DA signaling-dependent motor paralysis. Here, we demonstrate that *swip-10* mutations induce premature and progressive DA neuron degeneration, with light and electron microscopy studies demonstrating the presence of dystrophic dendritic processes, as well as shrunken and/or missing cell soma. As with paralysis, DA neuron degeneration in *swip-10* mutants is rescued by glial-specific, but not DA neuron-specific expression of wildtype *swip-10*, consistent with a cell non-autonomous mechanism. Genetic studies implicate the vesicular Glu transporter VGLU-3 and the cystine/Glu exchanger homolog AAT-1 as potential sources of Glu signaling supporting DA neuron degeneration. Degeneration can be significantly suppressed by mutations in the Ca^2+^ permeable Glu receptors, *nmr-2* and *glr-1*, in genes that support intracellular Ca^2+^ signaling and Ca^2+^-dependent proteolysis, as well as genes involved in apoptotic cell death. Our studies suggest that Glu stimulation of nematode DA neurons in early larval stages, without the protective actions of SWIP-10, contributes to insults that ultimately drive DA neuron degeneration. The *swip-10* model may provide an efficient platform for the identification of molecular mechanisms that enhance risk for Parkinson’s disease and/or the identification of agents that can limit neurodegenerative disease progression.

## Author summary

Glutamate (Glu) is an important signaling molecule used by nerve cells to communicate information, although excessive Glu signaling can overexcite neurons to the point where they degenerate, a phenomenon termed excitotoxicity. Glu induced excitotoxicity has been linked to neurodegeneration arising in the context of stroke, amyotrophic lateral sclerosis (ALS) and Parkinson’s disease (PD). Glial cells, that surround neurons, and their processes have been shown to limit Glu-induced excitotoxicity in mammals. Here, we demonstrate that *C*. *elegans* glia limit progressive degeneration of dopamine (DA) neurons that arises in the context of mutation of the protein, SWIP-10, and that this degenerative process relies on Glu signaling, altered Ca^2+^ homeostasis and apoptotic pathway genes. Our findings reveal a novel molecular contributor to glial maintenance of DA neuron viability, provide a genetically-tractable example of Glu-dependent cell death, and encourage further evaluation of SWIP-10 linked pathways for mechanistic insights into neurodegenerative diseases and their treatment.

## Introduction

Across phylogeny, the amino acid glutamate (Glu) plays multiple, important roles including contributions to protein synthesis, intermediary metabolism, and chemical neurotransmission [1–4]. At neuronal synapses, Glu signals through both metabotropic receptors that initiate G-protein coupled signaling [5–7] as well as ionotropic receptors that flux ions such as Na^+^ and Ca^2+^, altering membrane excitability [5, 8–10]. Excessive ionotropic Glu signaling in the mammalian brain has been implicated in a variety of brain disorders including addiction, schizophrenia, amyotrophic lateral sclerosis (ALS), and Parkinson’s disease (PD) [11–14], as well as the neuronal death that arises in the context of stroke and glioblastoma [15, 16]. Acute treatment of neurons with high, non-physiological, levels of Glu can induce signs of cell death within minutes, characterized by intense vacuolization and cell swelling characteristic of necrosis [17–20]. Chronic hyper-activation of neurons by Glu, within physiological limits, can drive apoptotic mediated neural degeneration, particularly if other genetic or environmental risk pathways are engaged [21–23]. Glu activation of Glu receptors can lead to prolonged alterations in intracellular Ca^2+^ homeostasis, driving Ca^2+^-dependent proteolysis and activation of apoptotic programs [24].

Although cell autonomous mechanisms remain a focal point for many investigations seeking insights into determinants of neurodegeneration, increasing attention has been given to astrocytic mechanisms that can sustain neuronal viability, in the context of constant Glu stimulation that could otherwise lead to cell death. These mechanisms include the shuttling of metabolic intermediates such as lactate to neurons that can help sustain ATP synthesis [25–27], the buffering of extracellular ions such as K^+^, since excess extracellular K^+^ due to chronic ion channel activation and Na^+^/K^+^ ATPase dysregulation can contribute to excess neuronal activation [26, 28, 29], and the efficient clearance of extracellular Glu that both limits the amplitude of synaptic and extrasynaptic Glu signaling but also Glu-driven neuronal degeneration [26, 30, 31]. Astrocytic Glu clearance is mediated by multiple Na^+^-dependent Glu-transporters of the SLC1 family (e.g. GLT1/rodents, EAAT2/humans) that terminate Glu signaling via binding and uptake of Glu in proximity to synaptic release sites [13, 31, 32]. A second astrocytic Glu transporter that participates in extracellular Glu homeostasis is xCT (SLC7A11), the transporter subunit of a dimer that supports intracellular Glu exchange for extracellular cystine. xCT is generally thought to act oppositely to SLC1 transporters, balancing control of extrasynaptic Glu levels with the provision of precursor (cysteine) for astrocytic glutathione synthesis [33–36].

Due to their significant impact on synaptic and extrasynaptic Glu homeostasis, Glu transporters and exchangers have been widely studied to determine their contribution to Glu-induced neural degeneration as well as in efforts to manipulate their activity and expression for therapeutic ends [36–38]. For example, Rothstein and coworkers identified β-lactam antibiotics, typified by the cephalosporin-type agent ceftriaxone (Cef), as capable of elevating GLT1 expression *in vitro* and *in vivo*, protecting neurons from Glu toxicity, and enhancing longevity in an ALS mouse model [13]. Subsequently, many investigators have demonstrated the neuroprotective activity of Cef administration in rodents [39–41], with evidence supporting antibiotic modulation of both GLT1 and xCT expression [13, 36, 42], although candidates targeted by the antibiotic in glia to induce transporter expression have, until recently, been unidentified.

In a screen for novel genes that control DA signaling in the nematode, *C*. *elegans* [43], we identified a glial-expressed gene, *swip-10*, whose mutation induces hyper-excitability of DA neurons and elevates rates of vesicular DA release, culminating in the hyperdopaminergic phenotype, Swimming induced paralysis (Swip) [44]. These studies also demonstrated a critical role for Glu signaling in establishing the paralytic phenotype of *swip-10* mutants [44]. *Swip-10* is conserved across phylogeny, with the unstudied gene, *Mblac1*, as the putative mammalian ortholog. Both SWIP-10 and MBLAC1 proteins are metallo β-lactamase domain (MBD)-containing proteins [44], with residues key for metal binding and catalysis conserved across worm and vertebrate proteins. Although the substrate hydrolyzed by SWIP-10/MBLAC1 enzymatic activity is currently unknown, we recently established that MBLAC1 is a specific, high-affinity target for Cef [45]. We presented evidence that Cef binding activity in brain lysates could be totally eliminated by MBLAC1 immunodepletion therefore supporting the hypothesis that MBLAC1 may be the exclusive, non-microbial target of Cef *in vivo*. These findings also suggest that further study of SWIP-10/MBLAC1 may reveal mechanisms normally engaged to protect neurons from chronically elevated extracellular Glu and a path to the identification of novel neuroprotective agents. A key piece of data lacking in this hypothesis, however, is evidence that loss of SWIP-10/MBLAC1 either induces Glu-dependent neural degeneration or eliminates the neuroprotective actions of Cef.

Here, we capitalize on the ease of monitoring the morphology and degeneration of *C*. *elegans* DA neurons engineered to stably express green fluorescent protein (GFP) to examine a requirement for *swip-10* expression in limiting Glu-dependent DA neuron degeneration. We find that *swip-10* mutants demonstrate a striking, progressive degeneration of DA neurons that can be suppressed by glial expression of wild type *swip-10* and by mutation of Ca^2+^ permeable Glu receptor mutants. Through our studies, we provide evidence that a cell non-autonomous action of SWIP-10 sustains DA neuron viability in the context of excess Glu signaling and elevations of cytosolic Ca^2+^ that we hypothesize leads to increased cellular stress and, ultimately, apoptotic cell death. Our findings support SWIP-10 (and by extension MBLAC1) as a key protective agent whose further study may yield important insights into risk factors for progressive neurodegenerative disorders and their treatment.

## Results

### Dopamine neuron degeneration observed in loss of function *swip-10* alleles

**Fig 1:**
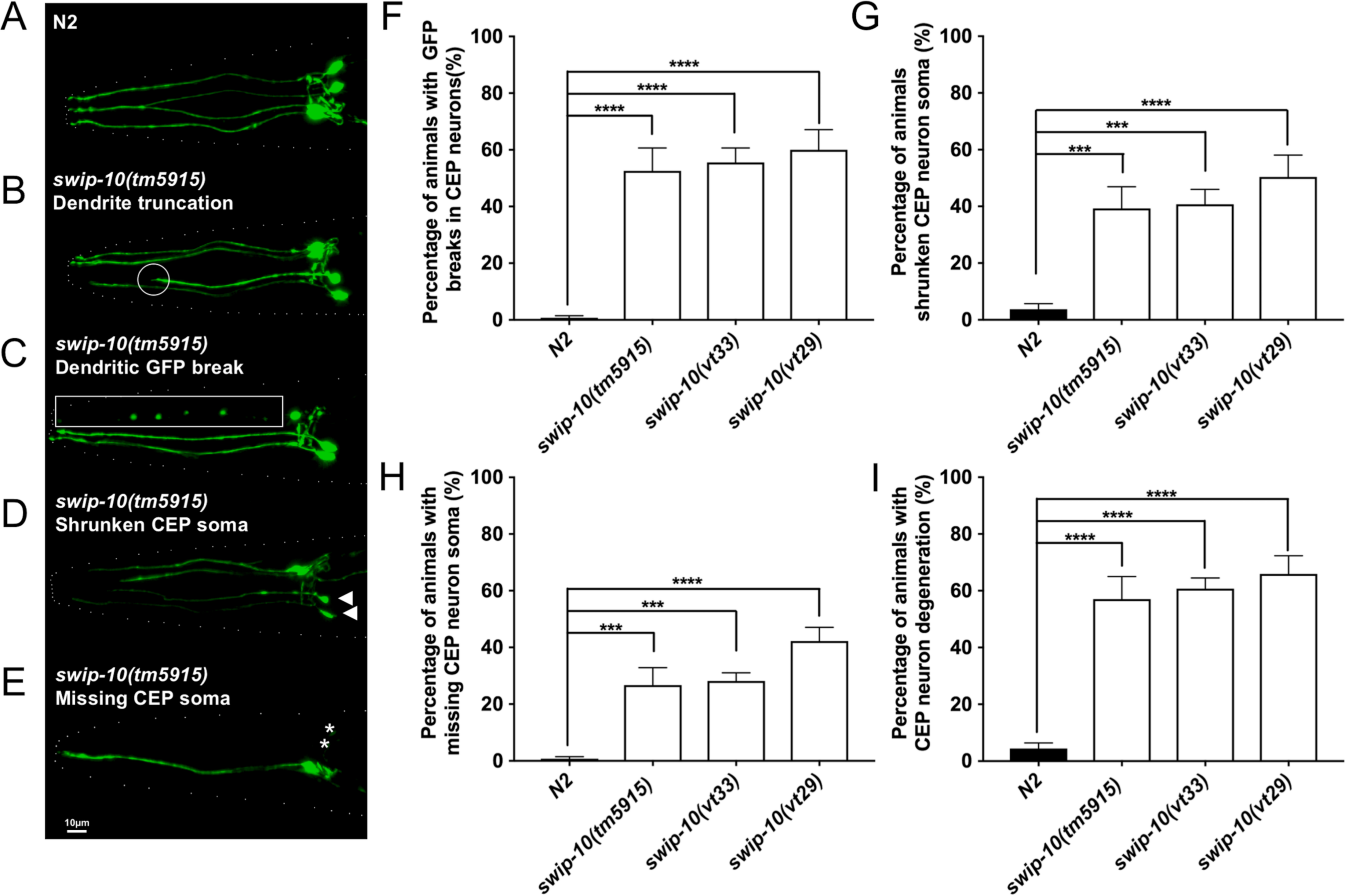
DA neuron degeneration observed in loss of function *swip-10* alleles. (A) N2 DA neurons labeled with GFP demonstrating evenly expressed fluorescence throughout the neuronal processes. (B-E) Representative images of *swip-10(tm5915)* mutant degeneration depicting (B) truncated CEP DA neuron dendrites indicated by a white circle, (C) breaks in GFP along CEP dendrites (white rectangle), (D) shrunken CEP cell soma (white arrowheads) and (E) missing CEP cell soma (white asterisks), scale bar is 10μm. (F-I) Quantification of the components of CEP DA neuron degeneration for (F) truncations/breaks in GFP, (G) shrunken CEP soma, (H) missing CEP soma, and (I) total degeneration phenotype, inclusive of all three degeneration measures. Data were analyzed by one-way ANOVA with Sidak’s post-test to N2; *** and **** indicate *P*<0.001 and 0.0001 respectively. Error bars represent ± SEM, with n = 105–150 animals per strain.

**Fig 2:**
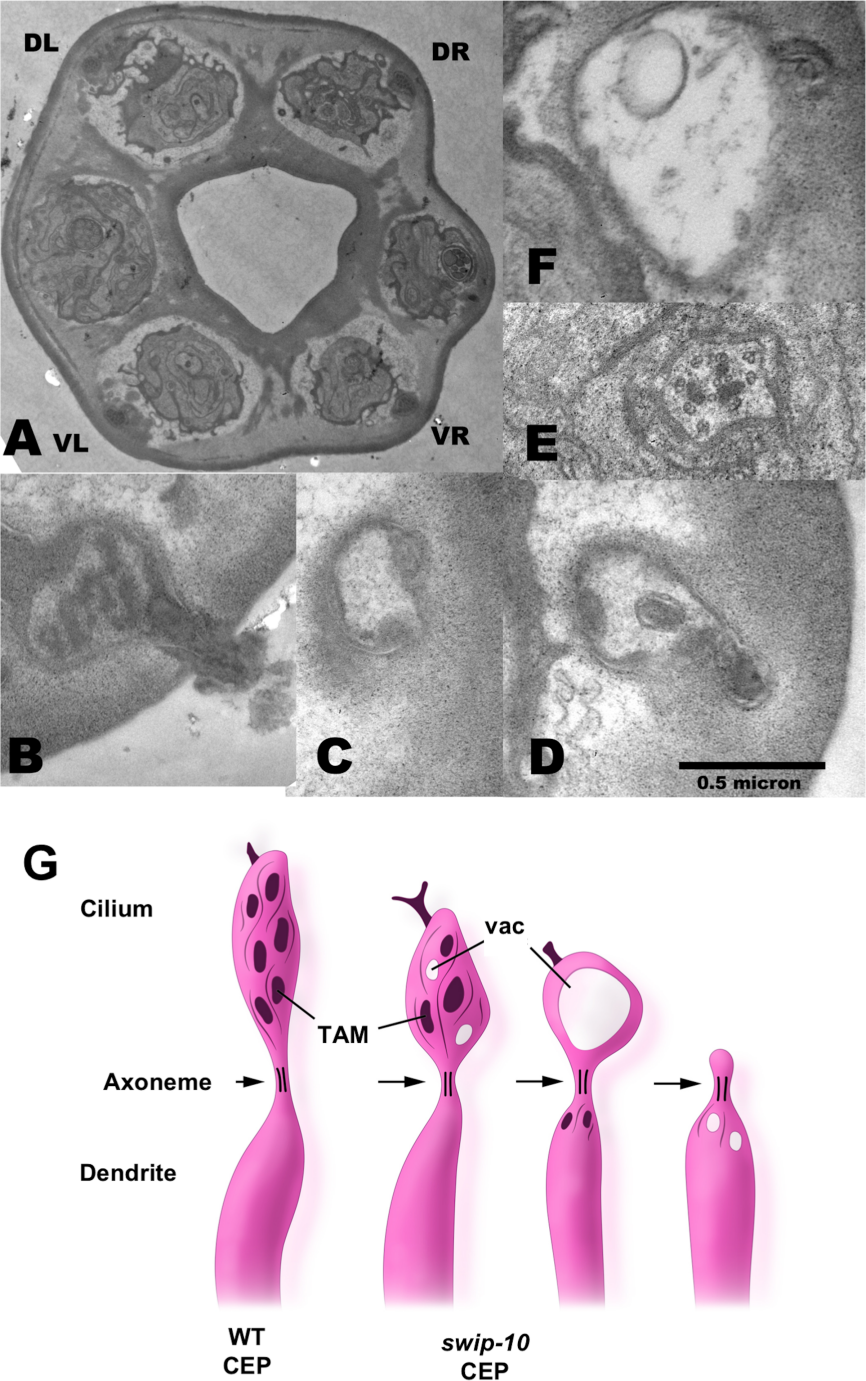
Electron microscopy confirms missing and deformed cilia of the CEP DA neuron dendrites in *swip-10* mutants. (A) Thin section through the lips of a *swip-10* mutant adult all four CEP cilia are formed almost normally in positions DL, DR, VL, VR, forming specialized endings embedded in the lip cuticle. (B) High power TEM image of *swip-10* mutant CEPVR cilium, somewhat reduced in overall size, containing normal-looking microtubules and dark staining tubule-associated material (TAM). The nubbin is abnormally enlarged and emerges out of the cuticle in an enlarged tree-like structure, not seen in N2. (C) Midway and more (D) distal through another the defective CEPVR cilium in a different animal, lacking normal TAM or distal microtubules. (E) and (F) show thin sections from a CEPDR cilium where small bits of TAM have abnormally become stuck inside the well-formed axoneme, while beyond the axoneme (F) the malformed cilium consists of large vacuole-filled swelling with no TAM or microtubules, and only a minimal nubbin. Scale bar (0.5 micron) applies to panels B-F. (G) Summary illustration of the variety of distal defects found in the CEP cilium, comparing a wildtype cilium to the left, and three progressively more defective mutant cilia on the right.

**Fig 3:**
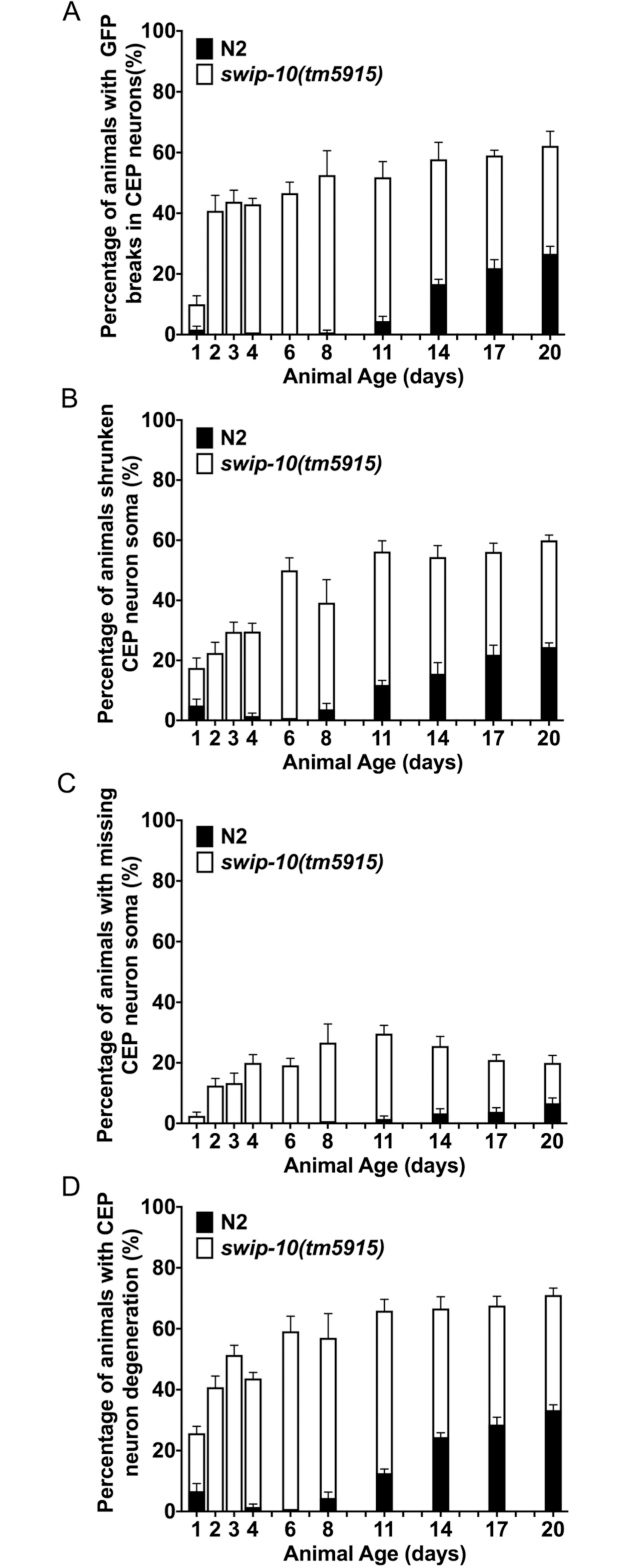
DA neuron degeneration increases with age, and *swip-10* mutant animals display earlier and more progressive levels of DA neuron degeneration. (A) *swip-10(tm5915)* mutants display dendritic breaks/truncations early in development, increasing in frequency with age. (B) *swip-10(tm5915)* mutants display shrunken soma earlier in development, increasing in frequency with age compared to N2. (C) *swip-10(tm5915)* mutants are missing soma earlier in development, increasing in frequency with age compared to N2. (D) As assessed by the combined degeneration index, both N2 and *swip-10* DA neuron degeneration increases with age, though the *swip-10* mutant DA neurons display degeneration at earlier ages than N2. Analyzed by two-way ANOVA with significant differences by age (**** *P*<0.0001) and by genotype (**** *P*<0.0001) and significantly different age by genotype interaction (**** *P*<0.0001). Error bars represent ± SEM, with n = 105–150 animals per strain per stage.

Given the Glu signaling-dependent, Swimming-induced paralysis (Swip) phenotype present in *swip-10* mutants [44], and evidence from the latter study that *swip-10* DA neurons are hyper-excitable, as assessed by a cytoplasmic Ca^2+^ reporter (GCamp), we sought to determine whether these animals might display signs of excitotoxic neural degeneration. We examined the DA neurons of multiple mutant *swip-10* alleles crossed to BY250, a strain that stably expresses the integrated transcriptional fusion *p*_*dat-1*_::*GFP* (*vtIs7)* (Fig 1A) [46]. We focused our evaluations on CEP DA neurons, and quantitatively evaluated degeneration by three distinct morphological assessments: 1) neurite truncations and breaks in GFP-labeled dendrites (Fig 1B and 1C), 2) shrunken cell soma (Fig 1D) and 3) missing cell soma (Fig 1E), as previously described [47, 48]. From these categories, we also calculated an overall degeneration score where the appearance of any of the components qualifies an animal as displaying CEP degeneration [48]. We found that all three available *swip-10* alleles (*vt29* and *vt33* from our forward genetic screen, and the larger deletion allele, *tm5915*) exhibited elevations in the degeneration index, relative to wildtype animals (Fig 1F–1I). To further support that mutation of *swip-10* induces morphological changes in DA neurons, versus a sequestration or inactivation of cytoplasmic GFP, we corroborated our findings using a DA neuron-targeted, membrane-bound reporter (*p*_*dat-1*_::*myrRFP*) which also yielded evidence of *tm5915* DA neurodegeneration (S1 Fig). Interestingly, evaluation of *swip-10* impact on *C*. *elegans* glia broadly (marked by the *ptr-10* promoter driven myrRFP) or on CEPsh glia that ensheath CEP DA neurons specifically (marked by *p*_*hlh-17*_::GFP) failed to reveal evidence for gross morphological changes (S2 Fig). These findings suggest that *swip-10* mutation induces a localized, cell non-autonomous effect on the integrity of neighboring DA neurons.

### Electron microscopy confirms DA neuron degeneration in *swip-10* mutants

To be sure that our fluorescent reporters of DA neuron morphology were faithfully reporting structural changes in DA neurons, we assessed CEP cilia of *swip-10* via electron microscopy (EM). Previously, we used this approach to document damage to CEP dendrites in the context of 6-OHDA induced DA neuron degeneration [49]. The *tm5915* deletion allele was selected for EM studies of *swip-10* induced neural degeneration, though as noted above, all mutants demonstrated comparable degeneration. The morphology of CEP neuronal processes is well characterized at the ultrastructural level [50], especially the specialized cilium at the tip of the CEP dendrite, which can be visualized in transverse thin sections through the lips of adult *C*. *elegans* (Fig 2A) [51, 52]. Using relative position and the defined morphological characteristics of CEP DA neurons, such as the electron dense cuticular branch or nubbin associated with their cilia to anchor the dendrite to the cuticle [52] and the presence of the electron dense clumps of tubule-associated material (TAM) previously shown to be characteristic of CEP cilium [51], we were able to identify multiple anomalies in *tm5915* CEP structure. These defects include changes in the size and appearance of the nubbin (Fig 2B), loss or misplacement of TAM and microtubules (Fig 2C and 2D), and the presence of large or small vacuoles in several locations either below or above the axoneme (Fig 2E and 2F). A summary of the *swip-10* mutant CEP cilium defects is depicted in Fig 2G. In addition to the defects described above, half of the CEP dendrites of *swip-10* mutants were missing any cilium that extended beyond the axoneme. These TEM studies confirm that *swip-10* mutation results in striking DA neuron morphological changes.

### Dopamine neuron degeneration increases with age

In order to determine whether the DA neuron degeneration observed in *swip-10* animals represents a late onset phenomenon and/or might arise from a progressive perturbation across development, we assayed DA neuron degeneration in *swip-10* mutants across various post-embryonic ages. We observed that *tm5915* animals display time-dependent indications of DA neuron degeneration that are distinct from the changes seen with wildtype animals (Fig 3D). In wildtype animals, signs of DA neuron degeneration are evident only in older, adult animals whereas signs of degeneration are already evident in *tm5915* animals by day 1 (L1 stage) of larval development (Fig 3D). A breakdown of the components that comprise the overall degeneration score of *tm5915* mutants is revealing, where non-uniform patterns are evident across measures. Although we were unable to follow individual DA neuron morphological changes over time, our population findings are suggestive of a progressive form of degeneration at the single neuron level, with dendritic breaks and truncations as earliest signs of degeneration (Fig 3A), followed by the appearance of shrunken soma (Fig 3B), and then by missing soma (Fig 3C). Overall similar patterns are evident with wildtype animals, just appearing much later in life. Together our findings indicate that *swip-10* mutation begins to disrupt the health of DA neurons early in development with the appearance of indices of morphological perturbations arising in distal processes that progress to neuronal death.

### Mutation of glial-expressed *swip-10* drives DA neuron degeneration

**Fig 4:**
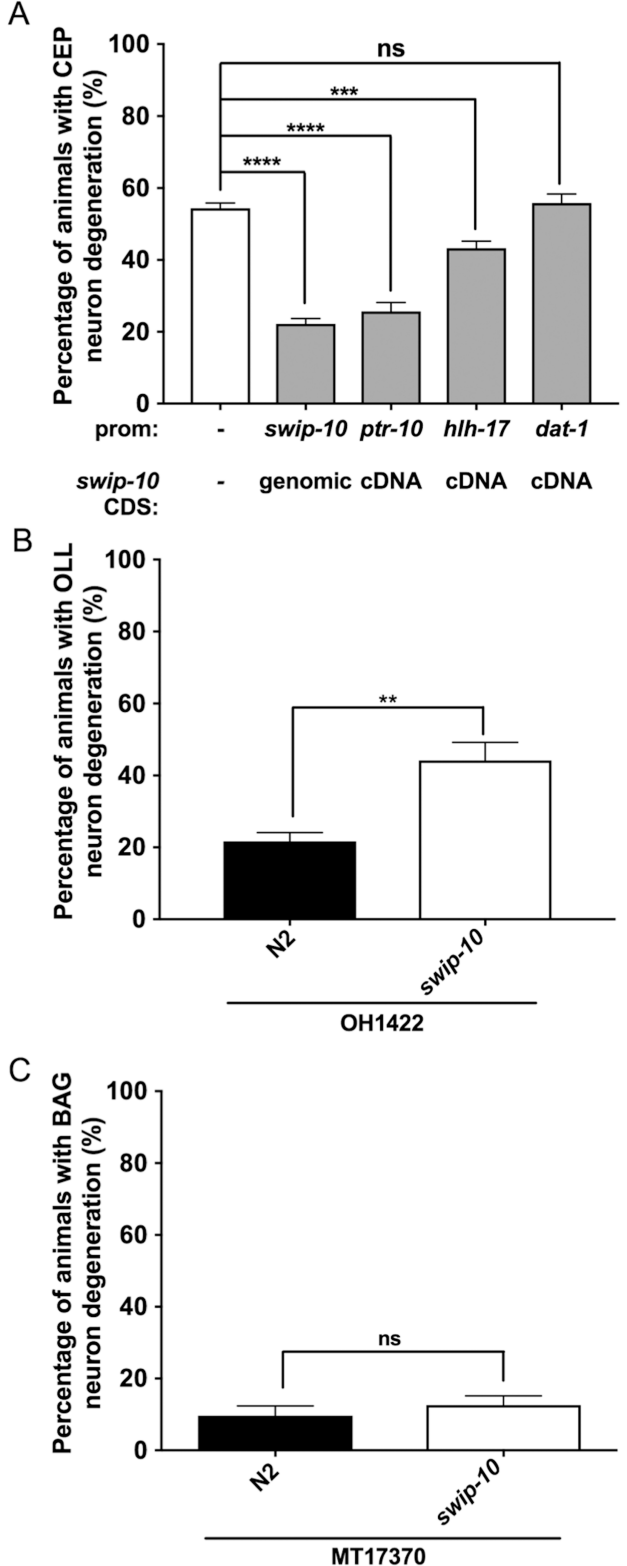
Glial expressed *swip-10* is required for normal DA neuron morphology, and glial ensheathment may be important for *swip-10* support of neuronal health. (A) Expression of *swip-10* genomic fragment significantly restores DA neuron morphology in *swip-10* mutants. Expression of *swip-10* cDNA under the control of a pan glial promoter, *ptr-10*, and not under a DA neuron specific promoter (*dat-1*), significantly reduces *swip-10* DA neurodegeneration, to similar levels as *swip-10* genomic rescue. Additionally, expression of *swip-10* cDNA in the CEPsh glial cell significantly reduces *swip-10* CEP DA neuron degeneration. Data were obtained comparing non-transgenic and transgenic progeny, assayed in parallel. (B) The OLL neurons of *swip-10* mutants display morphological characteristics similar to CEP neuron degeneration, including breaks in dendritic GFP, shrunken soma or missing soma (individual components not shown), and quantification of the three components together or “total degeneration” phenotype shows that OLL neurons in *swip-10* mutants are significantly different from N2. (C) The BAG neurons of *swip-10* mutants display normal N2 morphology as determined by quantification of total degeneration in gravid adult animals. The horizontal line beneath the genotypes on the x-axis refers to the background strain, (B) OH1422, with an integrated OLL neuron transcriptional reporter *(wgIs328*[P_ser2prom3_::GFP]) and (C) MT17310, with an integrated BAG neuron transcriptional reporter (*nsIs242*[_Pgcy-33_::GFP]). Data were analyzed using an unpaired Student’s *t* test (A-C), with **, ***, **** indicating *P*<0.01, <0.001, and <0.0001, respectively, ns = non-significant (*P*>.05), error bars represent ± SEM, with n = 105–150 animals per strain.

Although the Swip behavior of *swip-10* mutants at the L4 stage arises as a consequence of excess DA signaling [43], this paralysis is a cell non-autonomous consequence of glial, and not DA neuron, expression of *swip-10* [44]. To determine whether the degeneration of DA neurons is similarly a consequence of mutation of glial *swip-10*, we expressed a full length wild type *swip-10* cDNA fused to GFP (*swip-10*::GFP) under control of glial and DA neuron promotors. Fig 4A demonstrates that pan-glial *swip-10* expression, as achieved through use of the *ptr-10* promoter [53], robustly rescues DA neuron degeneration of *tm5915* animals, comparable to that achieved with a genomic construct that encodes *swip-10* and the upstream elements needed to achieve full rescue of Swip [44]. Significant rescue of DA neural degeneration was also achieved with the CEP sheath glia-specific promotor *hlh-17* [53]. In contrast, DA neuron specific expression of *swip-10*, driving expression with the *dat-1* promoter, failed to restore normal morphology. Together, these findings support the conclusion that glial expression of *swip-10* is required to maintain the normal viability of DA neurons.

### *swip-10* support for neuronal health may be related to glial ensheathment

Although not explored extensively, we sought to understand whether neural degeneration in *swip-10* mutant animals is limited to the DA neurons. We chose to evaluate *swip-10* mutant (*tm5915)* animals bearing reporters to demarcate OLL and BAG neurons. Glutamatergic OLL neurons are similar in location and morphology to dopaminergic CEP neurons, are mechanosensitive like CEP neurons, and share an association with glia (OLLsh) that ensheath OLL processes. Carbon-dioxide sensing, glutamatergic BAG neurons are similar in location and morphology to the CEP neurons, although not associated with direct ensheathing or socket glia. We observed degeneration of glutamatergic OLL neurons (Fig 4B) but not of BAG neurons (Fig 4C). These findings, along with rescue of DA neurodegeneration through glial re-expression of *swip-10*, reinforce a key role for glia in maintaining the viability of *C*. *elegans* DA neurons.

### Glu signaling contributes to DA neuron degeneration induced by *swip-10* mutation

Mechanisms proposed to support DA neuron degeneration include mishandling of intracellular DA that can form cytotoxic quinones [54, 55]. Thus, elevations in cytosolic DA that arise with pharmacological blockade of the vesicular monoamine transporter (VMAT, *cat-1* in *C*. *elegans)* by reserpine results in DA neuron degeneration [48], and a genetic reduction of VMAT2 expression causes progressive DA neuron degeneration in mammals [56]. The degeneration of DA neurons in *swip-10* animals does not appear to arise as a consequence of elevations of intracellular DA as disruption of DA synthesis capacity arising from a loss of function mutation in *cat-2*, the *C*. *elegans* ortholog of tyrosine hydroxylase, the rate-limiting step in DA synthesis, did not alter *tm5915* DA neuron degeneration (S3A Fig). Extracellular DA elevations can lead to the formation of toxic adducts with vital cell proteins [57] and our prior studies support excess DA secretion in swip-10 animals [44]. However, loss of extracellular DA clearance capacity achieved via mutation of the presynaptic DA transporter, *dat-1*, which triggers Swip, [58] did not induce DA neuron degeneration (S3B Fig).

**Fig 5:**
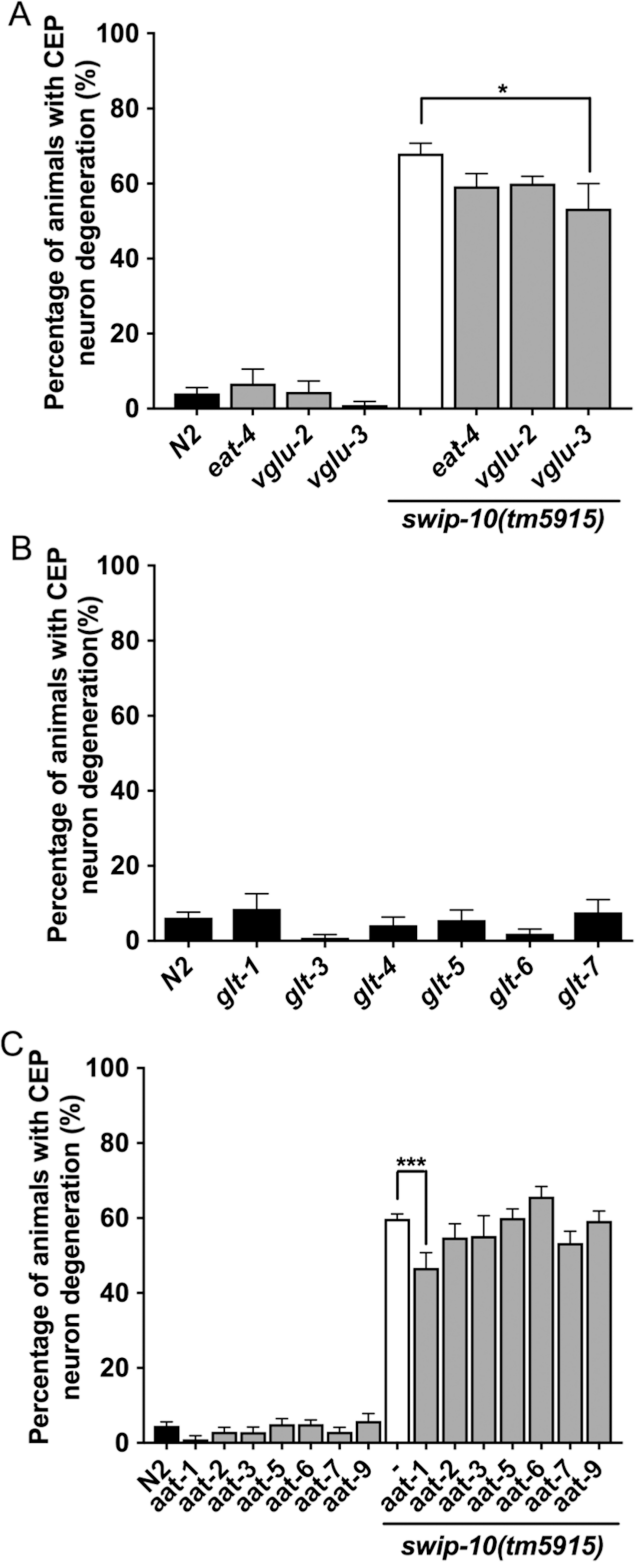
Disruption of Glu signaling attenuates the DA neuron degeneration of *swip-10* mutants. (A) Loss of the vesicular Glu transporter, *vglu-3*, suppresses *swip-10* DA neuron degeneration, whereas loss of *eat-4* or *vglu-2* does not significantly alter the levels of *swip-10* neurodegeneration. (B) Disrupting Glu clearance by loss of individual Glu transporters is not sufficient to induced DA neurodegeneration. (C) Loss of the amino acid transporter, *aat-1*, but not *aat-2*, *aat-3*, *aat-5*, *aat-6*, *aat-7 or aat-9*, significantly attenuates the DA neuron degeneration of *swip-10* mutants. Data were analyzed by a one-way ANOVA with Sidak’s post-tests, with * and *** indicating *P*<0.05 and <0.001 respectively, error bars represent ± SEM, with n = 105–150 animals per strain.

Neural degeneration, more generally, can be triggered by extrinsic or intrinsic activation of cell death genetic programs, first elucidated at a molecular level in *C*. *elegans* [59–62]. Additionally, disruptions of vital cellular processes (e.g. ATP production, membrane permeability, ion gradients or cytoskeletal organization) by genetically encoded neurotoxins or following exposure to reactive chemical species [63–65] have been shown to lead to the death of neurons. Lastly, excitotoxicity, a form of neurodegeneration with features of both apoptotic and necrotic cell death, is well known in mammalian brain preparations and typically observed in the context of over stimulation of Glu-responsive, ionotropic receptors [10, 65, 66]. Our prior findings that DA neurons in *swip-10* animals display elevated excitability that is dependent on Glu signaling [44] encouraged our consideration of the latter mechanism of DA neuron degeneration. We therefore quantified DA morphological changes in *swip-10* animals bearing loss of function mutations in genes supporting synaptic Glu packaging and Glu signaling, as well as mutations in genes encoding transporters thought to modulate extracellular Glu levels. First, we examined contributions of vesicular Glu transporters (vGLUTs). These proteins are responsible for packaging Glu into synaptic vesicles prior to release [67, 68]. There are three genes that encode proteins homologous to VGLUTs in *C*. *elegans* (*eat-4*, *vglu-2* and *vglu-3)* [69–71] with *eat-4* being the only one functionally characterized to date [68, 72]. Loss of individual vGLUTs (Fig 5A) had no effect on DA neuron morphology. Interestingly, whereas *eat-4* mutation significantly reduced the Swip behavior of *swip-10* mutants [44], the same *eat-4* allele failed to blunt the degeneration of DA neurons in *tm5915* animals. *vglu-2* mutation was also unable to reduce DA neuron degeneration. In contrast, loss of *vglu-3* significantly, suppressed DA neuron degeneration (Fig 5A), suggesting a contribution of vesicular Glu signaling, directly or indirectly, to *swip-10* DA neuron degeneration.

Mammalian glia express multiple Na^+^-dependent Glu transporters (GLTs) of the SLC1 family that support efficient clearance of Glu after release at synapses and their dysfunction figures prominently in investigations of Glu-dependent neuronal injury and death [31]. Additionally, our previous studies [44] demonstrated that mutation of several GLTs (*glt1*, *glt3* and *glt4*) conferred DA-dependent Swip. However, we found that mutation of individual *glt* genes failed to induce DA neuron degeneration (Fig 5B).

A second, glial Glu transport system, xCT, regulates extra-synaptic Glu levels, acting as a cystine/Glu exchanger [36]. xCT imports extracellular cystine in exchange for intracellular Glu, and thus altering the expression or activity of this transporter can modulate extracellular Glu levels. xCT is a member of the mammalian heteromeric amino acid transporter (HAT) family, for which there are 9 *C*. *elegans* homologs, with the highest homology for xCT being to AAT-1 and AAT-3 [73]. To determine whether xCT-like proteins could contribute to DA neuron degeneration, we generated *tm5915* double mutants with all available *aat* mutants. Of the 7 xCT homologs tested, we found that loss of *aat-1* uniquely attenuated the DA neuron degeneration of *tm5915* (Fig 5C). These findings implicate non-vesicular Glu release as a contributor to *swip-10* DA neuron degeneration. To determine if both vesicular Glu release supported by VGLU-3 and transporter-mediated Glu release supported by AAT-1 act in parallel or via a shared pathway to support DA neuron degeneration, we examined DA neuron morphology in an *aat-1*;*vglu-3* double mutant. We found no enhancement of the suppression of the *tm5915* degeneration beyond that of the individual mutants (S4 Fig). These findings are consistent with common mechanisms, downstream of extracellular Glu availability through either vesicular or non-vesicular Glu secretion mechanisms, as determinant of the quantitative extent of *swip-10* DA neuron degeneration.

**Fig 6:**
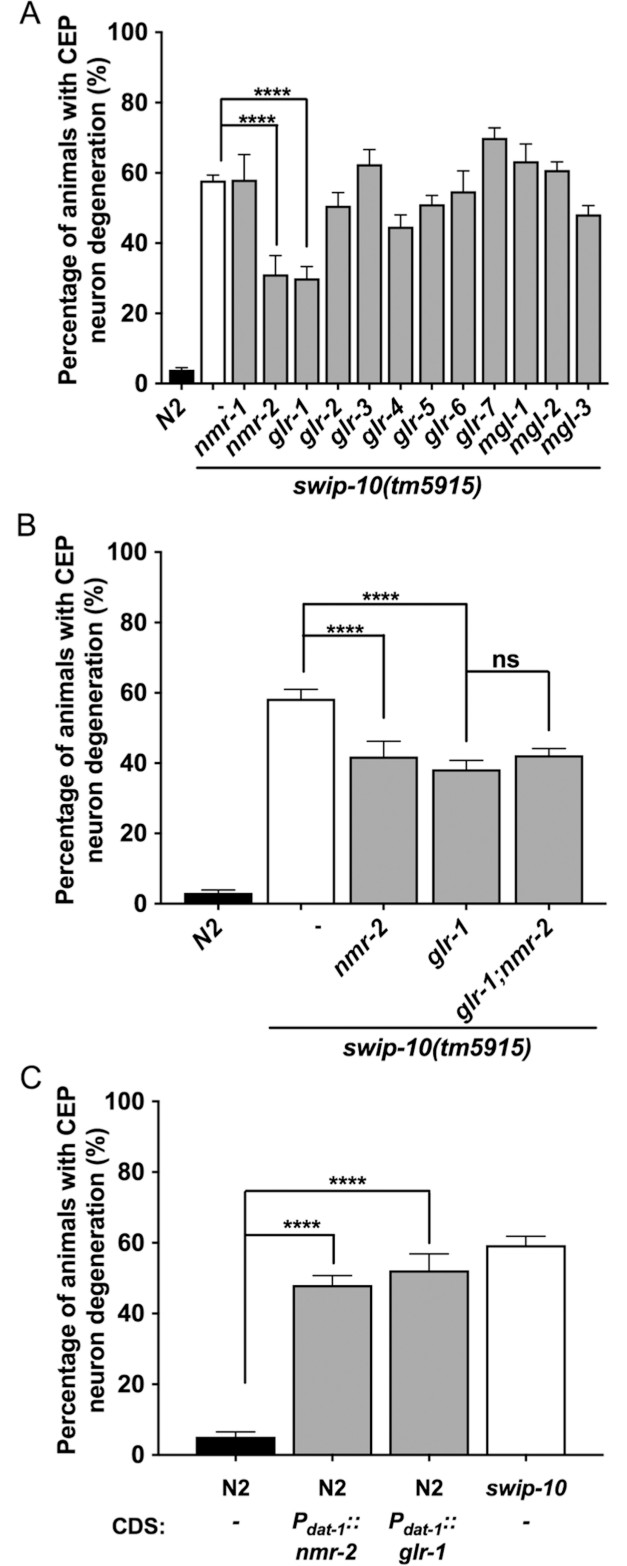
Support of DA neuron degeneration of *swip-10* mutants by ionotropic Glu receptor signaling and induction of DA neuron degeneration by DA neuron-specific *nmr-2* and *glr-1* overexpression. (A) Loss of the Ca^2+^-permeable ionotropic Glu receptors, *nmr-2* and *glr-1*, suppress *swip-10* mutant DA neuron degeneration. (B) Combinatorial loss of both *nmr-2* and *glr-1* does not suppress *swip-10* neurodegeneration beyond the suppression achieved by individual iGluR loss. (C) DA neuron-specific overexpression of either *nmr-2* or *glr-1* induces DA neuron degeneration in N2 animals. Analyzed by one-way ANOVA with Sidak’s post-tests (A, B) or unpaired Student’s *t* test comparing non-transgenic and transgenic progeny, assayed in parallel (C). **** indicates *P*<0.0001 and ns = non-significant (*P*>.05), error bars represent ± SEM, with n = 105–150 animals per strain.

Post-synaptically in both vertebrates and nematodes, Glu binds and activates ionotropic and metabotropic receptors (iGluRs and mGluRs, respectively) [74, 75]. To further pursue the hypothesis that mutation of *swip-10* triggers DA neuron degeneration via excess Glu signaling, we examined DA neuron morphology in *tm5915* lines bearing available mutant alleles for the iGluRs and mGluRs. Among the twelve GluR mutants tested, we found that loss of either the NMDA-type iGluR, *nmr-2*, or loss of the AMPA-type iGluR, *glr-1* [76], significantly suppressed *swip-10* DA neuron degeneration (Fig 6A). Interestingly, these GluRs are distinct from the GluRs previously shown to suppress the paralysis phenotype of *swip-10* mutants (*glr-4*, *glr-6* and *mgl-1)* [44]. A double mutant of *glr-1* and *nmr-2* did not further suppress *tm5915* degeneration beyond that seen with either mutant alone, suggesting that these receptors support neurodegeneration through a common pathway (Fig 6B). To further substantiate that excess GluR signaling via NMR-2 and GLR-1 could support our *swip-10* observations, we selectively overexpressed these receptors in DA neurons and examined CEP neuron morphology. As hypothesized, we detected statistically significant DA neuron degeneration, as compared to non-transgenic lines, similar to that observed in *swip-10* mutants (Fig 6C).

### Contributions of changes in intracellular Ca^2+^ to *swip-10* induced DA neuron degeneration

**Fig 7:**
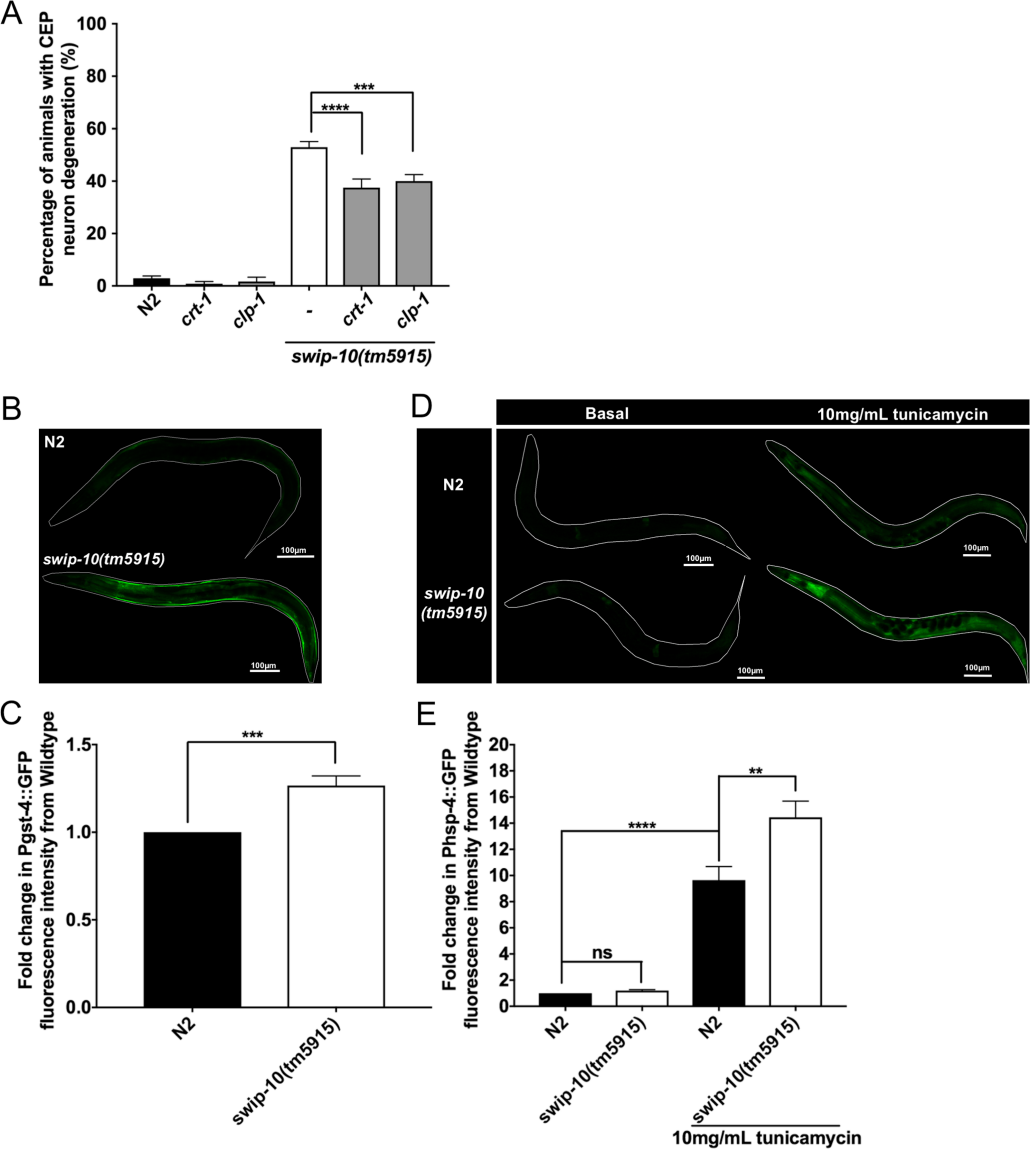
Contributions of changes in intracellular Ca^2+^ to *swip-10* induced DA neuron degeneration and activation of stress pathways. (A) Calreticulin (*crt-1*) and calpain-1 (*clp-*1) mutations suppress *swip-10(tm5915)* induced DA neurodegeneration. (B) Representative images of basal whole body oxidative stress reporter, *dvIs19*[*p*_*gst-4*_:GFP], fluorescence for N2 and *swip-10* gravid adult animals. Scale bar is 100μm. (C) Normalized reporter fluorescent intensity quantification reveals a significant increase in basal fluorescence in *swip-10* mutants. (D) Representative images of gravid adult, whole body ER stress reporter, *zcIs4*[*p*_*hsp-4*_:GFP]. Fluorescence for basal N2 and *swip-10* animals and N2, and *swip-10* animals grown on NGM/OP50 plates with 10mg/mL tunicamycin are presented, scale bar is 100μm. (E) Normalized reporter fluorescent intensity quantification reveals no change in basal ER stress in *swip-10* mutants. Both N2 and *swip-10* animals significantly respond to the ER stressor, tunicamycin, with *swip-10* mutants significantly more sensitive to tunicamycin. Analyzed by one-way ANOVA and Sidak’s post-tests (A and E), or analyzed by Student’s *t* test (C), **, ***, and **** indicates a *P*<0.01 <0.001, and <0.0001 respectively, ns = non-significant (*P*>.05), error bars represent ± SEM, with n = 105–150 animals per strain.

The evidence presented above of a role of Ca^2+^-permeant iGluRs [77] in DA neuron degeneration, as well as our prior findings that *swip-10* DA neurons demonstrate an exaggerated Ca^2+^ elevation in response to food contact [44], suggested to us that DA neuron degeneration in these animals could reflect activation of Ca^2+^-dependent programs linked to apoptotic and/or necrotic cell death [78–80]. Consistent with this idea, we found that loss of the primary endoplasmic reticulum (ER) Ca^2+^ storage/binding protein, calreticulin (*crt-1)* protected against *swip-10* DA neuron degeneration (Fig 7A). Excessive activation of the Ca^2+^-activated protease calpain-1, has been shown to lead to cellular damage, including neurodegeneration, in both mammals and *C*. *elegans* [81–83]. In keeping with these findings, a loss of function mutation of *clp-1*, the *C*. *elegans* calpain-1 ortholog, significantly attenuated the DA neuron degeneration of *tm5915* animals (Fig 7A). Together, these results support the hypothesis that inappropriate or excessive elevations of intracellular Ca^2+^ support *swip-10* DA neurodegeneration. In mammals, aberrant excitotoxic Ca^2+^ signaling can generate reactive oxygen species (ROS) leading to activation of cell stress pathways that drive neuronal cell death [84, 85]. To explore this idea, we inspected *swip-10* animals for signs of oxidative stress by monitoring reporter expression from *P*_*gst-4*_::GFP. The *gst-4* gene encodes a glutathione-s-transferase, and is a target for the ROS responsive transcriptional regulator SKN-1(*C*. *elegans* Nrf2 ortholog) [86, 87]. As shown in Fig 7B and 7C, *tm5915* animals demonstrate a significant elevation in *P*_*gst-4*_::GFP expression. As a measure of ER stress, we monitored the transcriptional reporter, P_*hsp-4*_:GFP [88, 89]. Although *tm5915* animals did not show indications of basal ER stress with this marker (Fig 7D and 7E), they were more sensitive to the pharmacological ER stressor, tunicamycin, compared to N2 animals (Fig 7D and 7E).

### Evidence of an apoptotic pathway in *swip-10* DA neuron degeneration

**Fig 8:**
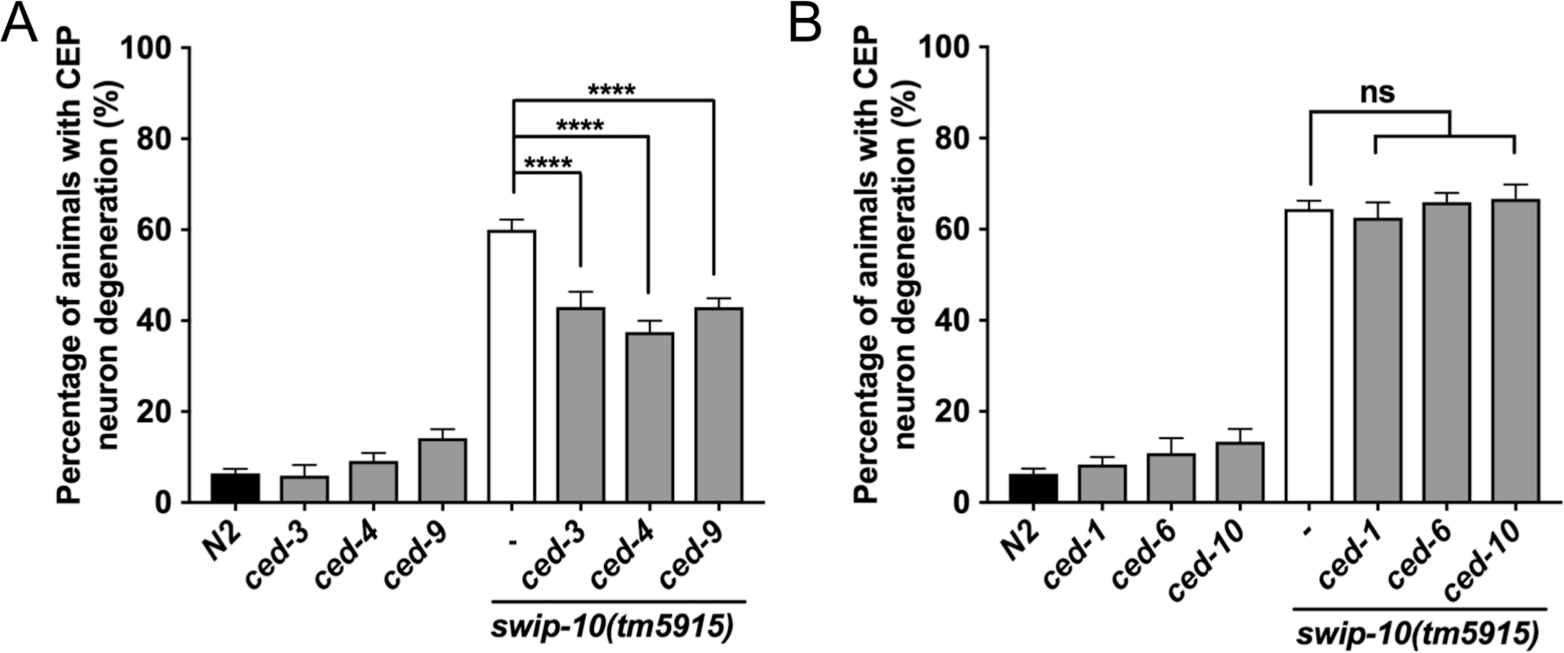
Genetic evidence for involvement of an apoptotic cell death program underlying DA neuron degeneration in *swip-10* animals. (A) Genetic disruption of the apoptotic cell death pathway, via loss of *ced-3* or *ced-4* or gain of function mutation to *ced-9* significantly reduces *swip-10* mutant CEP DA neuron degeneration. (B) Genetic disruption of genes involved in cell-corpse engulfment had no effect on the observed DA neuron degeneration of *swip-10* mutants. Analyzed by one-way ANOVA with Sidak’s post-tests (A and B), **** indicates *P*<0.0001, and ns = non-significant (*P*>.05), error bars represent ± SEM, with n = 105–150 animals per strain.

Glu-induced excitotoxicity has been reported to arise from multiple mechanisms, including necrosis, autophagy, and apoptosis [90], processes that also contribute to cell death in the nematode [61]. Cells dying by necrosis exhibit cell swelling and vacuolization [61], which we do not observe in *swip-10* animals (S5 Fig). In contrast, as described in Fig 1, DA neurons in *swip-10* mutants display blebbing or breaks in processes (Fig 1C and 1F) and shrunken soma (Fig 1D and 1G), features characteristic of apoptosis [91]. Consistent with this idea, we found that gain of function *ced-9* mutant animals [92] and loss of function *ced-4* and *ced-3* mutants, well-known contributors to programmed cell death [59], significantly suppressed *tm5915* DA neuron degeneration (Fig 8A). Apoptosis in the context of normal developmental programmed cell death is tightly coupled to cell corpse engulfment [93], with two partially-redundant and parallel pathways involving *ced-1/ced-6* [94, 95] and *ced-10* [96] responsible for recognition of dying cells and initiation of cell corpse clearance. Little is known concerning the integration of death and engulfment programs in relation to DA neuron cell death, though Offenburger recently reported contributions from both *ced-*6 and *ced-10* linked engulfment mechanisms in 6-OHDA induced DA neuron degeneration [97]. In contrast, we found that genetic disruption of individual genes associated with *ced-1/ced-6* and *ced-10* had no effect on measures of *swip-10* DA neuron degeneration (Fig 8B). These findings suggest that *swip-10* DA neuron degeneration arises from the activation of a cell-autonomous apoptotic pathway, one that draws little observable support from known engulfment mechanisms.

## Discussion

**Fig 9:**
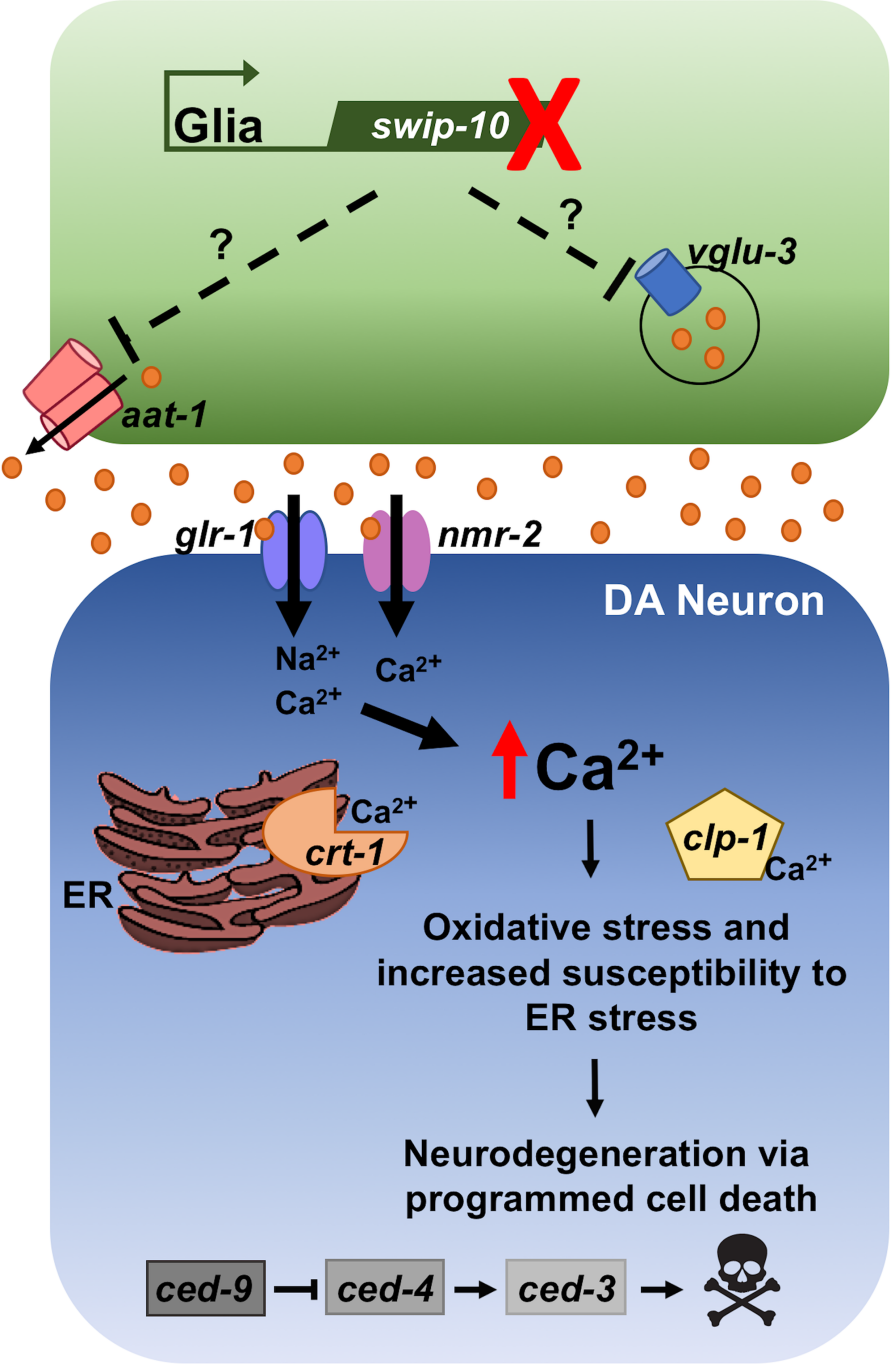
A suggested mechanism of *swip-10* Glu-induced excitotoxic DA neuron degeneration. Our findings are consistent with glial expressed *swip-10* leading to an elevation of extracellular Glu arising from changes in *aat-1* mediated Glu/Cys exchange or *vglu-3* mediated vesicular Glu release, resulting in the involvement of excess Glu activation of the Ca^2+^-permeable iGluRs, *nmr-2* and *glr-1*. Elevated tonic iGluR stimulation then drives pathological elevations in intracellular Ca^2+^-levels, increases cellular stress and activates apoptotic cell death pathways.

Overall, our findings reveal that loss of glial-expressed *swip-10* results in DA neuron degeneration through a process supported by excess Glu signaling through Ca^2+^-permeant ionotropic Glu receptors and Ca^2+^-dependent cell death mechanisms that engage apoptotic cell death pathways, as summarized in Fig 9. Although, we predominantly characterized *swip-10* DA neuron viability in gravid (egg-laying) adults, time-dependence studies indicate that degeneration is evident by day 2 post-hatching, and increases on all degeneration measures across the lifespan. A predominant display of fragmented or truncated dendrites in young animals versus shrunken or lost soma at later stages (Fig 3) suggests that degeneration in individual neurons is progressive, first emerging as altered neurite structure, followed by engagement of all compartments and eventually resulting in disappearance of some DA neurons altogether. This progressive pattern of degeneration is commonly seen with neurons suffering from energy depletion, that can be triggered by excessive stimulation or through metabolic poisoning [98, 99].

Most of our observations were obtained with a DA neuron-specific, cytosolic, fluorescent reporter, findings corroborated using a membrane anchored reporter (Figs 1 and S1). Subsequent studies of *swip-10* mutants using electron microscopy to image DA dendrites and cilia provided clear evidence of physical alterations (Fig 2) that we believe reflect the declining health of the DA neurons, versus a direct action of *swip-10* or its immediate effectors though further studies are needed. Additional EM studies would also be valuable in investigating the degeneration of *swip-10* mutants at the level of the DA neuron cell bodies, axons, and synapses. There are significant technical challenges associated with identifying these cells and processes in the densely populated nerve ring, though future studies that make use of correlated light electron microscopy (CLEM) can be envisaged [100].

The discovery that DA neurons degenerate in *swip-10* animals was initially surprising as our identification of *swip-10* derives from a hyperdopaminergic behavioral phenotype [44], though we previously demonstrated reduced DA levels in these animals [43]. Since *swip-10* DA neurons exhibit increased excitability and tonically-elevated DA secretion rates [44], we hypothesize that the degenerative process we have characterized likely contributes to a perturbation of mechanisms that insure a tight control over DA release (e.g. DA autoreceptors), along with a diminished capacity for DA clearance, leading to Swip. Alternatively, a degeneration-induced loss of DA signaling capacity could result in a hypersensitivity of postsynaptic DOP-3 DA receptors, such that DA release arising from water immersion then triggers excessive inhibition of motor neurons and Swip. In support of the latter possibility, movement of *swip-10* animals on plates reveals a heightened sensitivity to exogenous DA [43].

Having generated evidence for an age-dependent degenerative process impacting the morphology of *swip-10* DA neurons, we pursued mechanistic studies through a combination of genetic and imaging techniques. Such approaches have provided for a systematic elucidation of mechanisms underlying both programmed and environmentally-triggered cell death [59, 101–104]. In addition to the apoptotic pathways that drive programmed cell death during development, molecular determinants of later stage necrotic neuronal death, that arise as a result of the constitutive activity of mutant ion channels [105, 106] and ligand-gated Glu receptors [107], have been investigated. A potential role for excess Glu signaling in *swip-10* DA neuron degeneration seemed plausible given the contribution of Glu receptors and Glu transporters to Swip reported in our prior study [44]. In this regard, the groups of Driscoll and Mano have provided evidence that necrotic cell death arises with excess Glu signaling that occurs from a combined loss of Glu clearance and a hyperactive, constitutively active form of the alpha subunit of the G-protein, G_s_ [108–110]. Although *swip-10* DA neuron death shares features associated with the degeneration described in Mano’s studies, specifically the contributions from the iGluR, *glr-1* (Fig 6A), and the intracellular Ca^2+^ sequestering protein, *crt-1* (Fig 7A), our analysis also reveals a number of differences. Thus, besides a lack of morphological evidence of swollen, vacuolated soma seen in prior studies (S5 Fig), we found no evidence for a contribution to DA neuron degeneration of the adenyl cyclase ortholog, *acy-1*, nor could we implicate the autophagy-associated, cell death protein kinase, *dapk-1* (S6 Fig) [108, 109]. We also obtained evidence that the damaging effects of *swip-10* mutation are quite distinct from those observed with 6-OHDA induced DA neuron cell death. For example, the degeneration of DA neurons that arises within a day following 6-OHDA administration to wildtype worms lacks contributions from genes that participate in programmed cell death mechanisms [49], whereas, as we discuss below, contributions of these genes are evident in the *swip-10* model. Moreover, recent studies indicate that *ced-6* and *ced-10* dependent engulfment pathways support 6-OHDA induced loss of DA neurons [97], whereas we found no contribution of these engulfment genes to *swip-10* effects (Fig 8B). Moreover, Offenburger and colleagues have reported that 6-OHDA induced DA neuron death is exacerbated by mutation of the Ca^2+^ chaperone *crt-1* whereas we demonstrated *crt-1* mutation confers neuroprotection [111]. Together, these findings indicate that the DA neuron degeneration induced by *swip-10* mutation is an altogether unique form of neural degeneration as compared to prior glutamatergic and exogenous neurotoxin models.

A critical step in defining the mechanism associated with *swip-10* DA neuron degeneration is to determine the site(s) where wildtype *swip-10* expression is required to support normal DA neuron morphology. As with the rescue of DA-dependent Swip behavior [44], we found that glial *swip-10* re-expression, both genomic and cDNA, rescued *swip-10* DA neuron degeneration, whereas DA neuron expression of *swip-10* was without effect. These findings attest to a cell non-autonomous mode of action and raise the possibility that glial loss of *swip-10* may damage the glial cells themselves, rendering them unable to engage in secretory or contact-mediated support for DA neurons. Although non-quantitative, we detected no obvious morphological differences between wildtype and *swip-10* glia (S2 Fig), which may indicate that *swip-10* expressing glia are deficient in a capacity to provide specific trophic or metabolic support to DA neurons, versus participating in critical cell autonomous mechanisms. Future studies using higher resolution, EM-based methods should be pursued to refine this analysis. Importantly, we obtained rescue of *swip-10* DA neuron degeneration with a promoter driving wildtype *swip-10* expression in CEP sheath glia. Moreover, degeneration was apparent in OLL neurons that like CEPs are ensheathed by glia but not in BAG neurons, which lack these contacts. These studies reinforce a contribution of glia to the cell non-autonomous actions of *swip-10* to sustain neuronal viability and suggest that neurons in close apposition to ensheathing glia may preferentially depend on the activity of *swip-10*.

Our prior studies [44] assessing Swip behavior in *swip-10* mutants provided evidence of perturbed glial control of extracellular Glu that we hypothesized was responsible for the iGluR and mGluR dependence of Swip in these animals. We therefore considered the possibility that perturbed buffering of extracellular Glu by *swip-10* glia also underlies DA neuron degeneration. Mammalian literature emphasizes the critical role of glial Glu buffering mechanisms as protective against Glu excitotoxicity. As first described by Olney and colleagues, Glu excitotoxicity derives from excessive synaptic Glu acting on post-synaptic iGluRs [112–114], a process recapitulated by the actions of iGluR agonists such as kainic acid and ibotenate [115–117]. Moreover, inhibition of Glu transporters and increased extracellular Glu recapitulates the pathological hallmarks of PD in animal models, including DA neuron degeneration [118]. Our findings that mutations in the Ca^2+^ permeant iGluRs, *glr-1* and *nmr-2*, protect against *swip-10* DA neuron degeneration and that overexpression of these receptors leads to DA neuron degeneration in wildtype animals (Fig 6C) provide strong supportive evidence that glial mechanisms dictating the availability of extracellular Glu are likely disrupted in *swip-10* animals.

Mammalian glia have been reported to modulate extracellular Glu by vesicular release [119], Glu-permeable channels [120], synaptic clearance of Glu by Na^+^-coupled Glu transporters (GLTs) [31], and extrasynaptic Glu buffering by the cystine/Glu exchanger (xCT) [34, 35]. We found that a mutation in the vesicular Glu transporter *vglu-3* attenuates *swip-10* DA neuron degeneration (Fig 5A). We were surprised that an *eat-4* mutation did not contribute to *swip-10* induced degeneration, as such a mutation reduced Swip behavior [44]. Although the expression pattern and role for *vglu-3* is undetermined, these findings raise the possibility that EAT-4 supports Glu signaling in the neural circuitry that drives DA neuron excitation in response to water, whereas VGLU*-3* contributes to Glu release directly onto DA neurons, including SWIP-10 expressing glia, and drives tonic activation of Glu receptors on DA neurons and over time, excitotoxicity. Consistent with this model, distinct Glu receptors support Swip (GLR-4, GLR-6 and MGL-1) versus DA neurodegeneration (GLR-1 and NMR-2). Although we did not observe DA neurodegeneration with genetic loss of single GLT orthologs in the nematode (Fig 5B), unlike Swip [44], this may be due to genetic redundancy among the six GLTs. Indeed, studies by the Driscoll lab demonstrated that loss of one or two GLTs is insufficient to drive Glu-dependent neurodegeneration [121]. In contrast to our inability to implicate specific GLTs, we found that genetic disruption of the xCT related gene, *aat-1*, significantly reduced *swip-10* DA neuron degeneration (Fig 5C). As with *vglu-3*, the expression pattern for *aat-1* in the worm is undefined, and thus additional studies are needed to determine site(s) of expression that contribute to our results. The effects of *aat-1* mutation were not additive with those of *vglu-3*, suggesting that both genes act to support DA neurodegeneration through a common mechanism, which we propose is through the control of tonic, extracellular Glu providing tonic excitation of DA neuron expressed Glu receptors. Finally, it is important to note that mammalian xCT is upregulated by the β-lactam antibiotic ceftriaxone [36, 42, 122], which we have shown binds directly to the putative *swip-10* ortholog MBLAC1 [45]. Moreover, research, initiated by findings of Rothstein and colleagues [13], has demonstrated that ceftriaxone is neuroprotective, including in models of DA neuron degeneration [41].

Although not exclusive, Glu-induced neural degeneration often involves activation of Ca^2+^-permeable NMDA type iGluRs [19, 123, 124] and, as noted, our studies demonstrate an important contribution of Ca^2+^-permeable *C*. *elegans* iGluRs, the NMDA-type iGluR, *nmr-2*, as well as the AMPA-type iGluR, *glr-1* in *swip-10* neural degeneration[77] (Fig 6A). Expression profiling data provides evidence that *nmr-2* and *glr-1* are expressed in DA neurons [125]. Since *swip-10* mutant animals with loss of both *nmr-2* and *glr-1* do not demonstrate enhanced suppression of DA neural degeneration as compared to single receptor mutations (Fig 6B), we suggest that the flux of Ca^2+^ through one of these receptors is sufficient to increase intracellular Ca^2+^ sufficiently to initiate downstream signaling pathways that lead, over time, to neurodegeneration. Aberrant intracellular Ca^2+^ regulation and signaling has been implicated in excitotoxic cell death [126], with evidence supporting a role for Na^+^/Ca^2+^-permeable degenerin/epithelial sodium channels (DEG/ENaCs) [127–129], Ca^2+^-dependent proteases such as calpain [130, 131], and deficiencies in ER Ca^2+^ buffering [80, 106] in cell death mechanisms. We found that disrupting ER Ca^2+^ storage, by mutation of *crt-1*, or mutation of the *C*. *elegans* calpain ortholog, *clp-1*, significantly rescued *swip-10* DA neural degeneration (Fig 7A). Ca^2+^ dysregulation following excessive Glu stimulation has also been shown to engender multiple indications of cell stress including oxidative stress and ER stress [132, 133], which *swip-10* mutants display.

Finally, although acute Glu excitotoxicity has been more typically associated with necrosis [17, 19], evidence suggests that chronic dysregulation of Glu signaling and altered intercellular Ca^2+^ homeostasis can lead to activation of apoptotic pathways [134, 135], and a recent study by Anilkumar and colleagues has demonstrated that external factors, such as nutrient availability, determine whether or not excess Glu signaling triggers apoptotic or necrotic cell death (Anilkumar 2017). Consistent with this idea, genetic disruption of apoptosis in *C*. *elegans* [59] significantly reduced the DA neurodegeneration of *swip-10* mutants (Fig 8A). The progressive DA neuron degeneration we detect in *swip-10* animals supports the occurrence of a chronic insult and thus is in line with our genetic findings of apoptotic program engagement. However, our data suggests that *swip-10* involvement of apoptotic cell death associated genes differs from the involvement of these genes in developmental programmed cell death, as loss of genes critical for cell-corpse engulfment during programmed cell death did not alter the levels of *swip-10* DA neuron degeneration (Fig 8B). Although lack of a reliance on engulfment genes could be a reflection of the partial redundancy of the two major engulfment pathways, we suspect that these findings are indicative of a slower engagement of apoptotic genes in the *swip-10* model. Additionally, the majority of our assays are conducted at a mid-point, with degeneration in progress, to capture various degrees of degeneration in *swip-10* animals, it is possible that we have simply not assessed the correct temporal window for engulfment.

Although we present clear evidence for a significant role of excess Glu signaling in the degeneration of *swip-10* DA neurons, other mechanisms besides changes in extracellular Glu homeostasis are likely to contribute to our observations since Glu homeostasis and signaling mutants afford incomplete suppression of *swip-10* DA neurodegeneration. The elucidation of the normal role and genetic pathway for wildtype *swip-10* in *C*. *elegans* glial cells will likely clarify other contributors to *swip-10* induced neural degeneration. For example, mammalian glia have been shown to support neurons by buffering ions such as potassium (K^+^) and hydrogen (H^+^) [136], and by providing metabolic support via lactate, glutathione, and ATP shuttling [26]. Although only limited data speaks to glial-neuronal crosstalk in worms, we suspect that one or more of these mechanisms contribute to the diminished viability of DA neurons in *swip-10* animals. As our transcriptional stress reporter data indicate a systemic increase in cellular stress mechanisms (Fig 7B–7E), it seems entirely likely that the perturbations induced by *swip-10* mutation extend beyond the deficits observed in CEP (and OLL) neuron viability. Since wholesale degeneration is not evident, we suspect that the premature degeneration of DA neurons reflects a more dependent relationship of these cells on glia. The selective loss of nigrostriatal DA neurons in idiopathic PD has been suggested to derive from an intrinsic vulnerability to stress, possibly arising from the reactivity of DA itself, as well as inefficient anti-oxidant protection, ultimately rendering these cells more vulnerable than others to Glu-induced cell death [137]. Since genetic elimination of the capacity to synthesize DA did not reduce *swip-1*0 DA neuron degeneration, we feel it more likely that excess Glu signaling drives degeneration in combination with a parallel loss of glial metabolic or trophic support required by DA neurons.

In summary, our findings reveal a previously unreported dependence of DA neurons on *C*. *elegans* glia, one that when disrupted leads to neuronal degeneration. DA degeneration triggered by glial loss of *swip-10* appears to be progressive and dependent on excess Glu signaling through Ca^2+^ permeant iGluRs. We propose that these effects lead to perturbed intracellular Ca^2+^ homeostasis and, progressively, the engagement of apoptotic cell death pathways. Our work adds support to studies in mammals that indicate a critical role of proper glial function in DA neuron viability [138–141] and reveals a new worm model of Glu excitotoxicity, one likely amenable to pharmacological manipulation that could provide insights to novel therapeutics to treat human neurodegenerative disorders.

## Materials and methods

### *C*. *elegans* strains

Strains were maintained as described previously [142]. We thank J. Rand (Oklahoma Medical Research Foundation); the *Caenhorhabditis* Genetics Center; Shohei Mitani of the National Bioresource Project at Tokyo Women’s Medical University; and Shai Shaham, Niels Ringstad, and Oliver Hobert for providing the strains used in this work. N2 (Bristol) served as our wild-type strain, and unless specified otherwise, we utilized the proposed null allele, TM5915, of *swip-10* [44]. Strains used in this study are enumerated per figure appearance in S1 Table.

### Plasmid construction and transgenic manipulations

In all cases, insertion of the DNA fragment of interest and the fidelity of the vector was confirmed by sequencing and all PCRs were performed using KAPA HiFi HotStart ReadyMix (Kapa Biosystems). All constructs resulted in C-terminal cDNA fusion to an *unc-54 3’* UTR. For the membrane bound transcriptional reporter, we used overlap PCR [143] and Gibson Assembly (NewEngland Biolabs) to subclone the 700bp *dat-*1 promotor into the myrRFP containing backbone from p_*ptr-10*_:myrRFP (gift from Shai Shaham) to create pRB1349 (p_*dat-1*_:myrRFP). For transgenic *swip-10cDNA*::*GFP* rescue experiments, DA neuron, pan-glial, and CEPsh glial expression was achieved using the previously described plasmids, pRB1157, pRB1158, and pRB1159, respectively [44]. Genomic full-length *swip-10* rescue experiments were conducted as previously described [44]. For the DA neuron-specific Glu receptor experiments, a PCR product (20ng/μL) was amplified by overlap PCR [143] to include the 700bp *dat-1* promoter and genomic *glr-1* from the ATG start to 2890 of genomic *nmr-2* from the ATG start to 2974 fused to *unc-54* 3’ UTR for injection, along with p_*unc-122*_:RFP (35ng/μL) and p_*dat-1*_:myrRFP (35ng/μL).

### Genetic crosses

Crosses were performed using publicly available, integrated fluorescent reporter strains to mark chromosomes *in trans*. Single worm PCR was performed to confirm the presence of the indicated mutation. For all deletions, we used a three primer multiplex strategy that produces PCR amplicons with a 100–200 bp difference between N2 and mutant. This method was highly effective in eliminating preferential amplification of a lower-molecular-weight species. In all cases, a synthetic heterozygous control was used to ensure that heterozygous clones could be identified. We identified recombinant lines by PCR genotyping of single worm genomic DNA lysates. All genotyping PCRs were performed with the KAPA Genotyping Kit (KAPA Biosystems). In some cases, alleles were sequenced with sequence-specific primers to verify mutation homozygosity (GeneHunter and EtonBioscience).

### Confocal imaging

Confocal microscopy of mutants on the BY250 strain background was performed using a Nikon A1R confocal microscope in the FAU Brain Institute Cell Imaging Core using a 20x or 60x oil-immersion objective and Nikon Elements capture software. Worms were immobilized using 30mM levamisole in M9 on a fresh 2% agarose pad and cover-slipped with a 1mm cover glass before sealing with paraffin wax [144].

### Neurodegeneration assay

The neurodegeneration assay was adapted from a previously described method [145]. In our case, we transferred 20 worms to normal NGM/OP50 plates as L4s and incubated these plates for 48hrs at 19°C until animals reached the gravid adult stage, unless otherwise noted. We then picked 15 worms into 20μL of 30mM levamisole in M9 on slides prepared with a 2% agarose pad. For imaging, we utilized a Zeiss Discovery V12 inverted fluorescent microscope outfitted with a Xenon UV light source and GFP/YFP/RFP filter sets. We used a Zeiss mono FWD 16mm objective lens to visualize Green Fluorescent Protein (GFP) containing integrated transgenes, *vtIs7[*P_dat-1_::GFP], *nsIs242*[_Pgcy-33_::GFP], *wgIs328*[P_ser2prom3_::GFP] selectively expressed in DA, BAG, and OLL neurons respectively, allowing us to examine neurodegeneration in a cell-specific manner. For the DA neurons, analysis was limited to CEP neurons, because out of the 8 DA neurons in *C*. *elegans*, the 4 CEP neurons display the clearest and most distinct dendritic projections and can be readily identified via both light and electron microscopy (see below). Neurons were examined for the presence of 1) breaks in the CEP dendrites 2) shrunken or 3) missing somas. Worms were counted as displaying degeneration if one or more of these features were present. Normal N2 CEP, BAG, and OLL neurons lacked any of these abnormalities at the gravid adult stage. Total animals with degeneration, shrunken and missing somas, or neurite breaks were calculated for each trial. The percentage of animals exhibiting each morphological trait was determined for graphical analysis. Animals were tested 15 animals/day on 7–9 separate days (n = 90–135 animals assayed per genotype) blinded to genotype.

### Electron microscopy

N2 and *swip-10* mutant animals were raised and maintained at 20°C on *E*. *coli* OP50/NGM plates and 2-day adult animals (fixed 2 days after the L4 stage) were fixed and embedded for transmission electron microscopy (TEM) following a chemical immersion protocol [146, 147]. Briefly, animals were first cut open in a cacodylate-buffered osmium tetroxide fixative, then *en bloc* stained in uranyl acetate, and dehydrated and embedded in Spurr resin. Thin sections were collected onto Formvar-coated slot grids and examined on a Philips CM10 electron microscope. Digital images were collected with an Olympus Morada camera on the TEM, and figures were created using Photoshop.

### Fluorescence microscopy with GFP stress reporters

All fluorescent stress reporter stains were a generous gift from Dr. Matt Gill (Scripps Research Institute, Jupiter, FL). All stress reporter strains were imaged as gravid adult animals grown at 19°C for 48hrs after transfer to a fresh OP50/NGM plate at the L4 stage. To determine levels of stress we used the transcriptional reporter strains, *dvIs19* [*p_gst-4_*:GFP] and *zcIs4* [*p_hsp-4_*:GFP] to measure oxidative stress and ER stress respectively. We adapted previously described methods [87, 88]. Briefly, the overall *p_gst-4_*:GFP fluorescence intensity/μm per 15–20 3 day adult *swip-10* animals and 15–20 3 day adult N2 animals (with subtracted background fluorescence per animal) was determined, and the fold change GFP intensity compared to N2 signal was calculated for all animals assayed from one population and subsequently averaged over 4 independent days (n = 60–75 animals assayed). As a positive control for oxidative stress, we picked 15–30 L4 N2 animals to OP50 plates 2mM paraquat (Sigma) mixed with the NGM agar [148]. *p_hsp-4_*:GFP fluorescence intensity/μm was assayed as described above. To determine susceptibility of *swip-10* mutants to ER stress, we transferred 15–30 L4 N2 and *swip-10* animals to NGM plates containing 10μg/mL tunicamycin (Sigma) [89]. For each of the stress reporters, images were acquired using identical imaging settings across blinded genotypes and drug treatments, via a Nikon A1R confocal microscope in the FAU Brain Institute Cell Imaging Core using a 4x objective and Nikon Elements capture and analysis software.

### Statistical analyses

All statistical tests were performed and graphs generated using Prism version 7.0. Data were analyzed by Student’s t-tests, one-way ANOVAs followed by Sudak or Dunnet’s post-hoc tests and two-way ANOVAs, where appropriate. A *P* < .05 was taken as evidence of statistical significance in all cases.

## Supporting information

S1 Table *C*. *elegans* strains used in the generation of data that appear in the figures. (XLSX)

S1 Fig Membrane-bound fluorescent reporter corroborates *swip-10* mutant DA neuron degeneration. Integrated vtIs7 [p_dat-1_:GFP] reporter in green and extrachromosomal p_dat-1_:myrRFP reporter in red show equal levels of degeneration in *swip-10* mutants. Representative images show normal (A) N2 DA neuron morphology, merged, and a representative image of a *swip-10* mutant animal (B) integrated marker, (C) extrachromosomal array marker and (D) merged, scale bar is 20μm. (E) DA neuron degeneration was quantified in animals expressing both DA neuron fluorescent reporters, and both demonstrate *swip-10* mutant animals have significantly increased DA neuron degeneration. Analyzed by Student’s *t* test, **** indicates a *P*<0.0001, error bars represent ± SEM, with n = 105–150 animals per strain.
(TIF)

S2 Fig *swip-10* mutants display normal glial morphology. Representative images of the CEPsh glia of N2 and *swip-10* mutant animals, crossed onto a strain bearing an integrated p_*hlh-17*_:GFP transgene (DCR1337, *nsIs105*). Scale bar is 10μm. Representative images of the glia of N2 and *swip-10* mutant animals, crossed onto a strain bearing an integrated p_*ptr-10*_::myrRFP transgene (nsIs108). Scale bar is 10μm.
(TIF)

S3 Fig The DA neuron degeneration of *swip-10* mutants is not a result of aberrant intracellular DA signaling or hyperdopaminergia. (A) Disruption of DA synthesis, by loss of the nematode tyrosine hydroxylase ortholog, *cat-2*, does not prevent the DA neuron degeneration of *swip-10* mutant animals. (B) Hyperdopaminergia, induced by disrupted DA clearance by loss of the DA transporter, *dat-1*, is not sufficient to induce DA neuron degeneration. Analyzed by Student’s *t* test, ns = non-significant (*P*>.05), error bars represent ± SEM, with n = 105–150 animals per strain.
(TIF)

S4 Fig Combinatorial loss of both *aat-1* and *vglu-3* suppresses *swip-10* neurodegeneration similarly to levels of suppression by individual *aat-1* loss. Data were analyzed by a one-way ANOVA with Sidak’s post-tests, ns = non-significant (*P*>.05), error bars represent ± SEM, with n = 105–150 animals per strain.
(TIF)

S5 Fig *swip-10* mutants do not display gross morphological characteristics of necrotic cell death. Single-plane phase contrast images merged with maximum intensity projection confocal image show the relative positions of the CEP dopamine neurons to the terminal bulb of the pharynx in (A) N2 and (E) *swip-10(tm5915)* animals. (B) and (F) show the maximum fluorescence intensity projection confocal image of N2 and *swip-10(tm5915)* animals respectively. (C) and (G) show the merged single plane phase contrast image and corresponding single plane GFP confocal image at a plane where 1 or more CEP cell soma are in focus for N2 and *swip-10(tm5915)* animals respectively. (D) and (H) show the single plane phase contrast images for N2 and *swip-10(tm5915)* animals respectively, demonstrating no visible vacuolated or altered cellular structures. Scale bar of 10 microns for A-H.
(TIF)

S6 Fig Mutation of genes previously implicated in Glu-dependent necrotic cell death do not alter *swip-10* induced DA neuron degeneration. Data were analyzed by one-way ANOVA with Sidak’s post-tests, ns = non-significant (*P*>.05), error bars represent ± SEM, with n = 105–150 animals per strain.
(TIF)
