# Supplementary figures and images for: Glial loss of the metallo β-lactamase domain containing protein, SWIP-10, induces age- and glutamate-signaling dependent, dopamine neuron degeneration
Source: PLoS Genet. 2018 Mar 28;14(3):e1007269. doi: 10.1371/journal.pgen.1007269 (PMC5891035; doi:10.1371/journal.pgen.1007269)

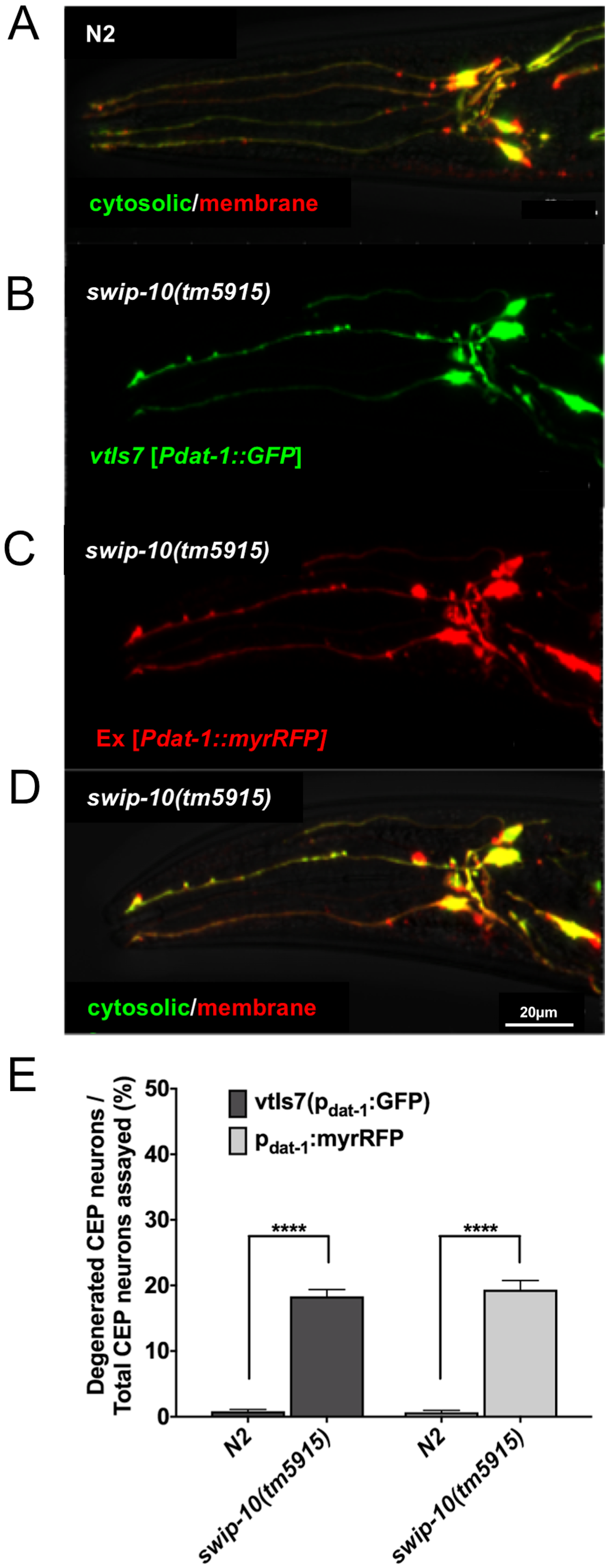

Supplement: S1 Fig — Integrated vtIs7 [pdat-1:GFP] reporter in green and extrachromosomal pdat-1:myrRFP reporter in red show equal levels of degeneration in swip-10 mutants. Representative images show normal (A) N2 DA neuron morphology, merged, and a representative image of a swip-10 mutant animal (B) integrated marker, (C) extrachromosomal array marker and (D) merged, scale bar is 20μm. (E) DA neuron degeneration was quantified in animals expressing both DA neuron fluorescent reporters, and both demonstrate swip-10 mutant animals have significantly increased DA neuron degeneration. Analyzed by Student’s t test, **** indicates a P<0.0001, error bars represent ± SEM, with n = 105–150 animals per strain. (TIF) [file pgen.1007269.s002.tif]

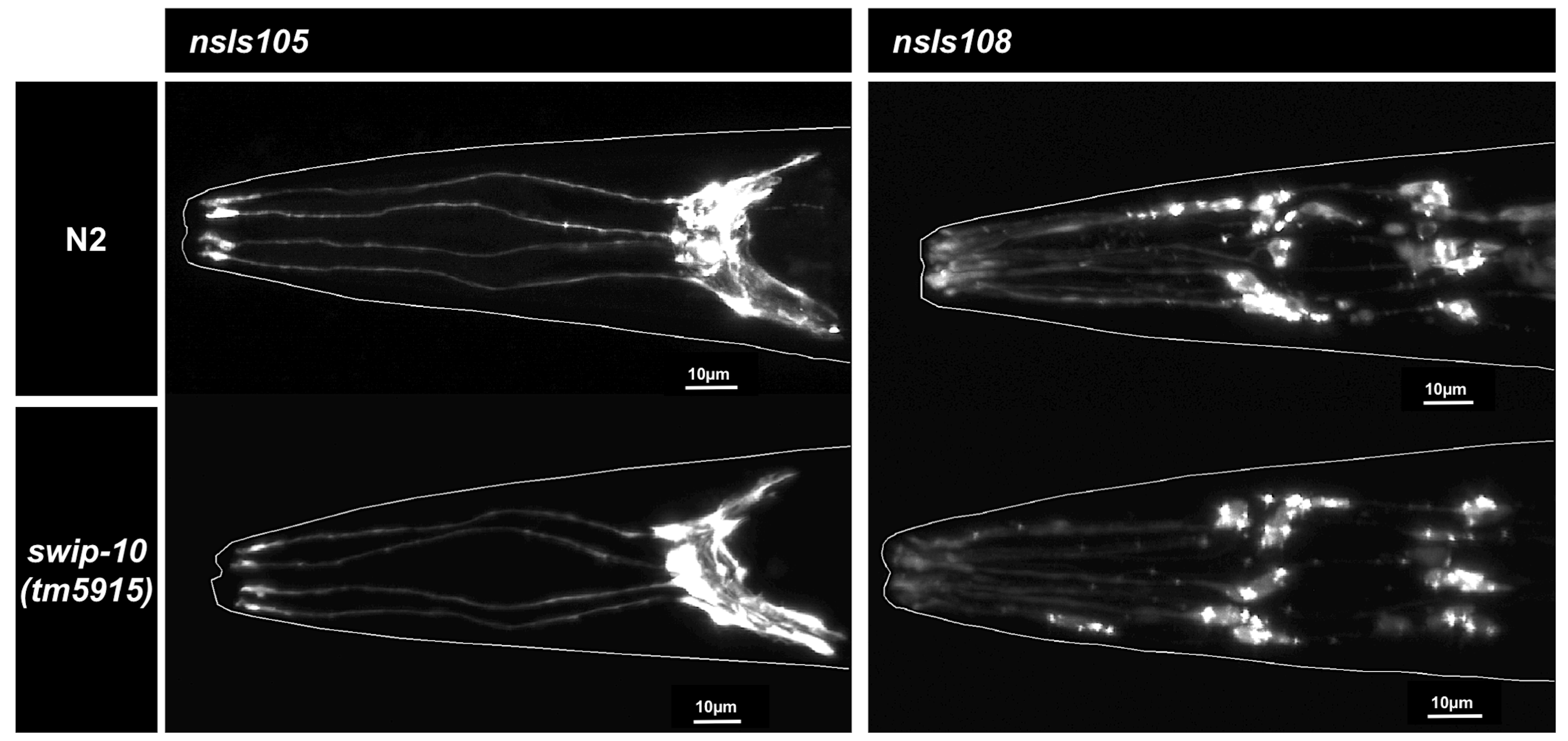

Supplement: S2 Fig — Representative images of the CEPsh glia of N2 and swip-10 mutant animals, crossed onto a strain bearing an integrated phlh-17:GFP transgene (DCR1337, nsIs105). Scale bar is 10μm. Representative images of the glia of N2 and swip-10 mutant animals, crossed onto a strain bearing an integrated pptr-10::myrRFP transgene (nsIs108). Scale bar is 10μm. (TIF) [file pgen.1007269.s003.tif]

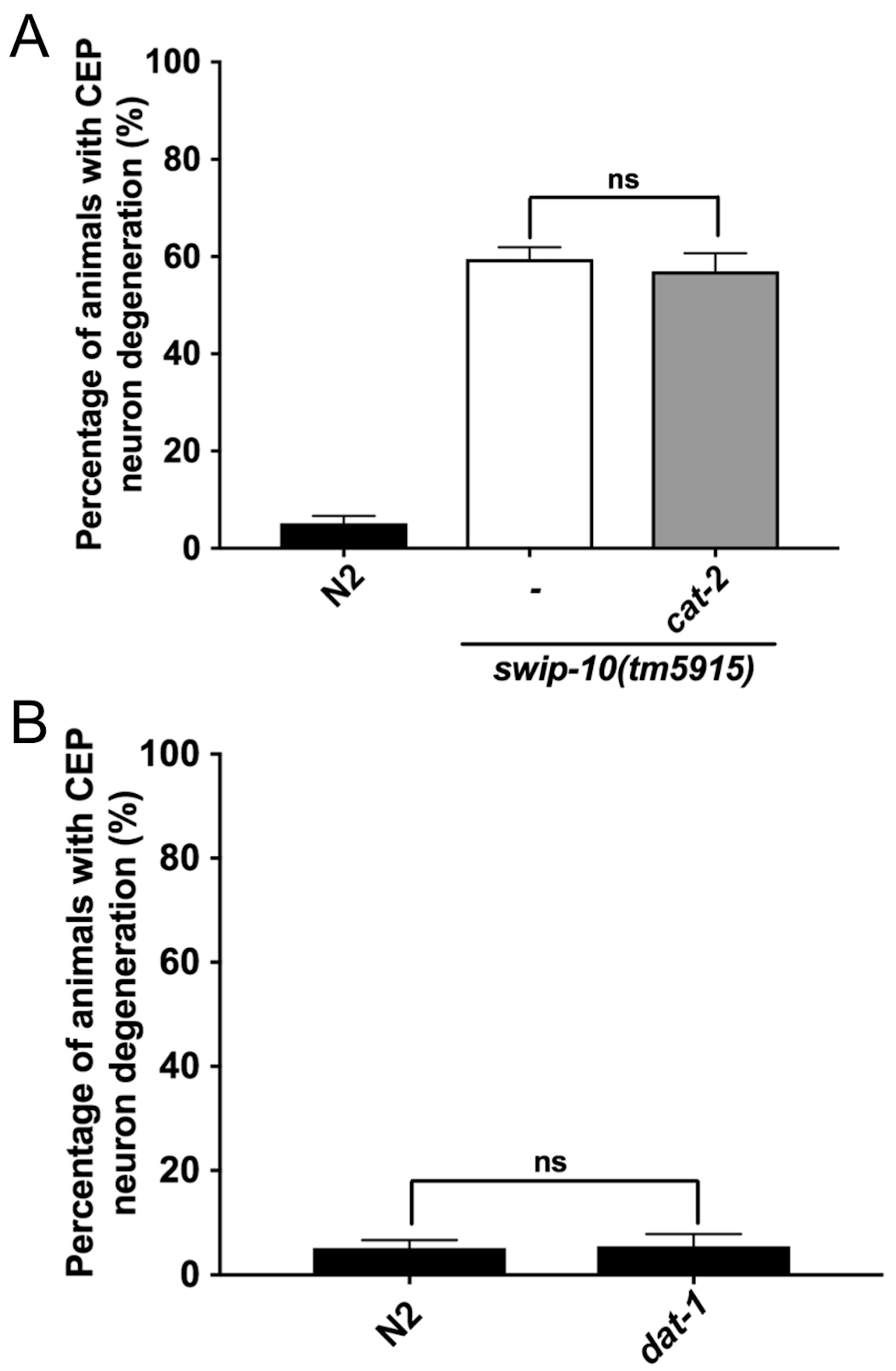

Supplement: S3 Fig — (A) Disruption of DA synthesis, by loss of the nematode tyrosine hydroxylase ortholog, cat-2, does not prevent the DA neuron degeneration of swip-10 mutant animals. (B) Hyperdopaminergia, induced by disrupted DA clearance by loss of the DA transporter, dat-1, is not sufficient to induce DA neuron degeneration. Analyzed by Student’s t test, ns = non-significant (P>.05), error bars represent ± SEM, with n = 105–150 animals per strain. (TIF) [file pgen.1007269.s004.tif]

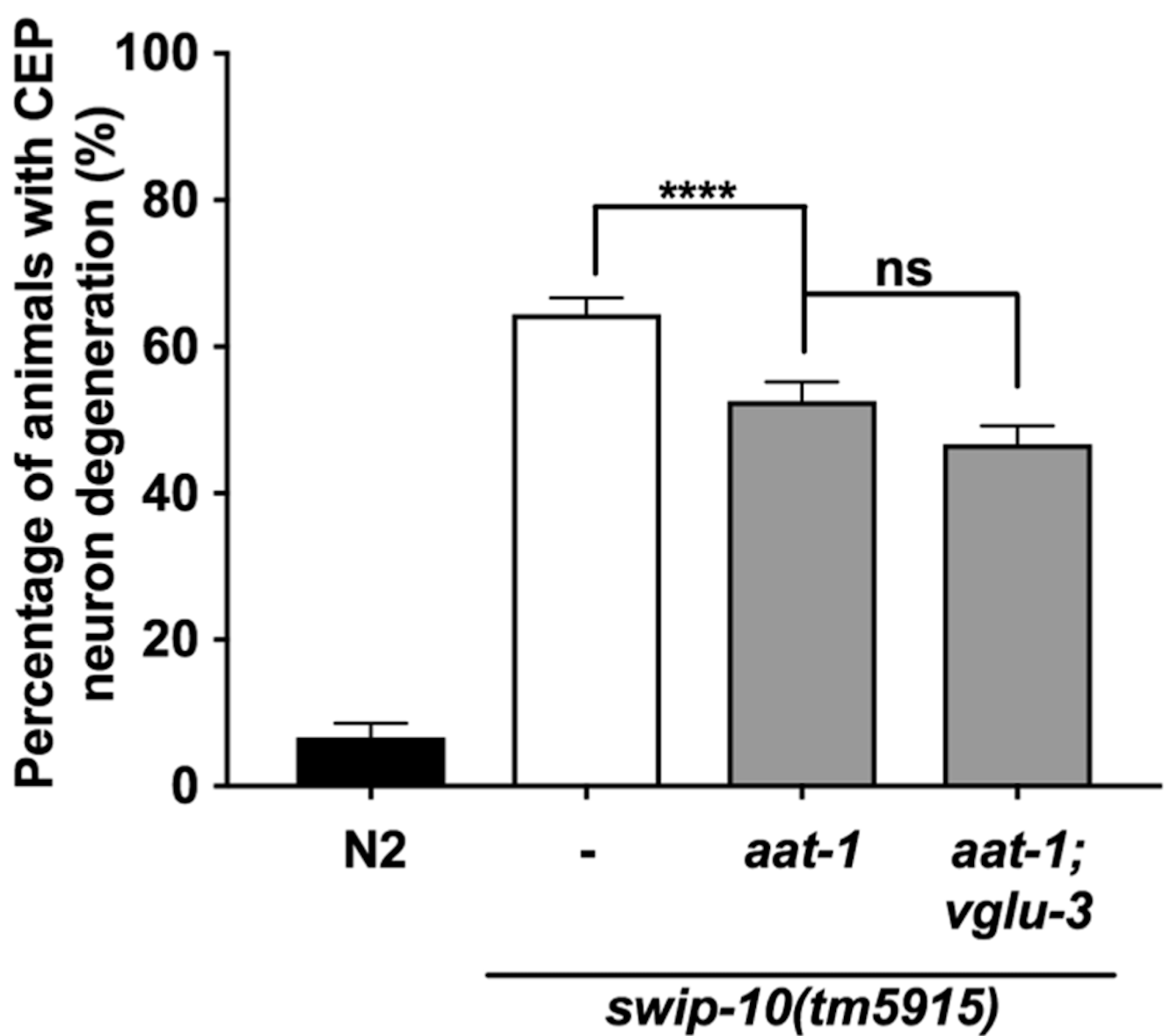

Supplement: S4 Fig — Data were analyzed by a one-way ANOVA with Sidak’s post-tests, ns = non-significant (P>.05), error bars represent ± SEM, with n = 105–150 animals per strain. (TIF) [file pgen.1007269.s005.tif]

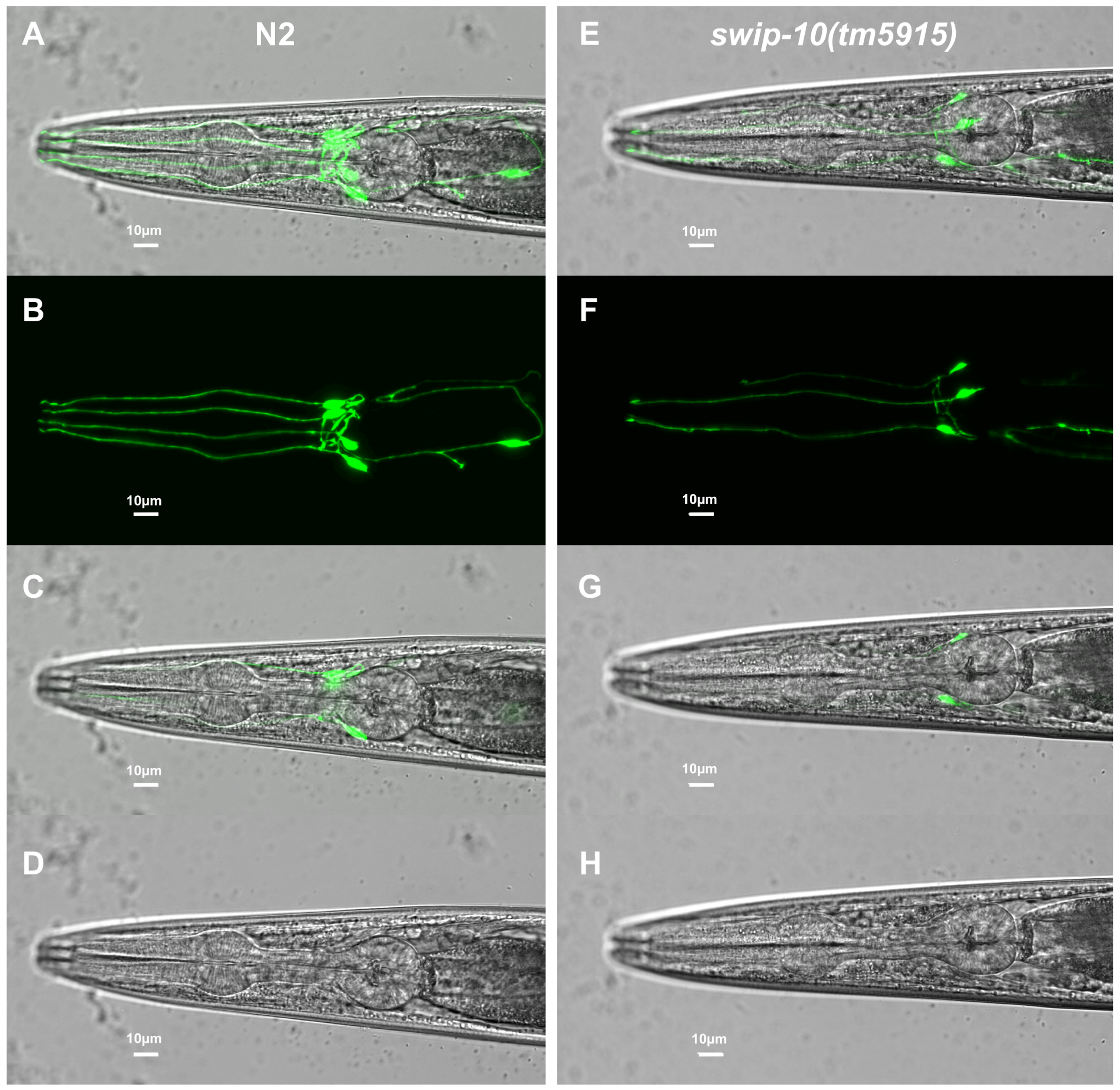

Supplement: S5 Fig — Single-plane phase contrast images merged with maximum intensity projection confocal image show the relative positions of the CEP dopamine neurons to the terminal bulb of the pharynx in (A) N2 and (E) swip-10(tm5915) animals. (B) and (F) show the maximum fluorescence intensity projection confocal image of N2 and swip-10(tm5915) animals respectively. (C) and (G) show the merged single plane phase contrast image and corresponding single plane GFP confocal image at a plane where 1 or more CEP cell soma are in focus for N2 and swip-10(tm5915) animals respectively. (D) and (H) show the single plane phase contrast images for N2 and swip-10(tm5915) animals respectively, demonstrating no visible vacuolated or altered cellular structures. Scale bar of 10 microns for A-H. (TIF) [file pgen.1007269.s006.tif]

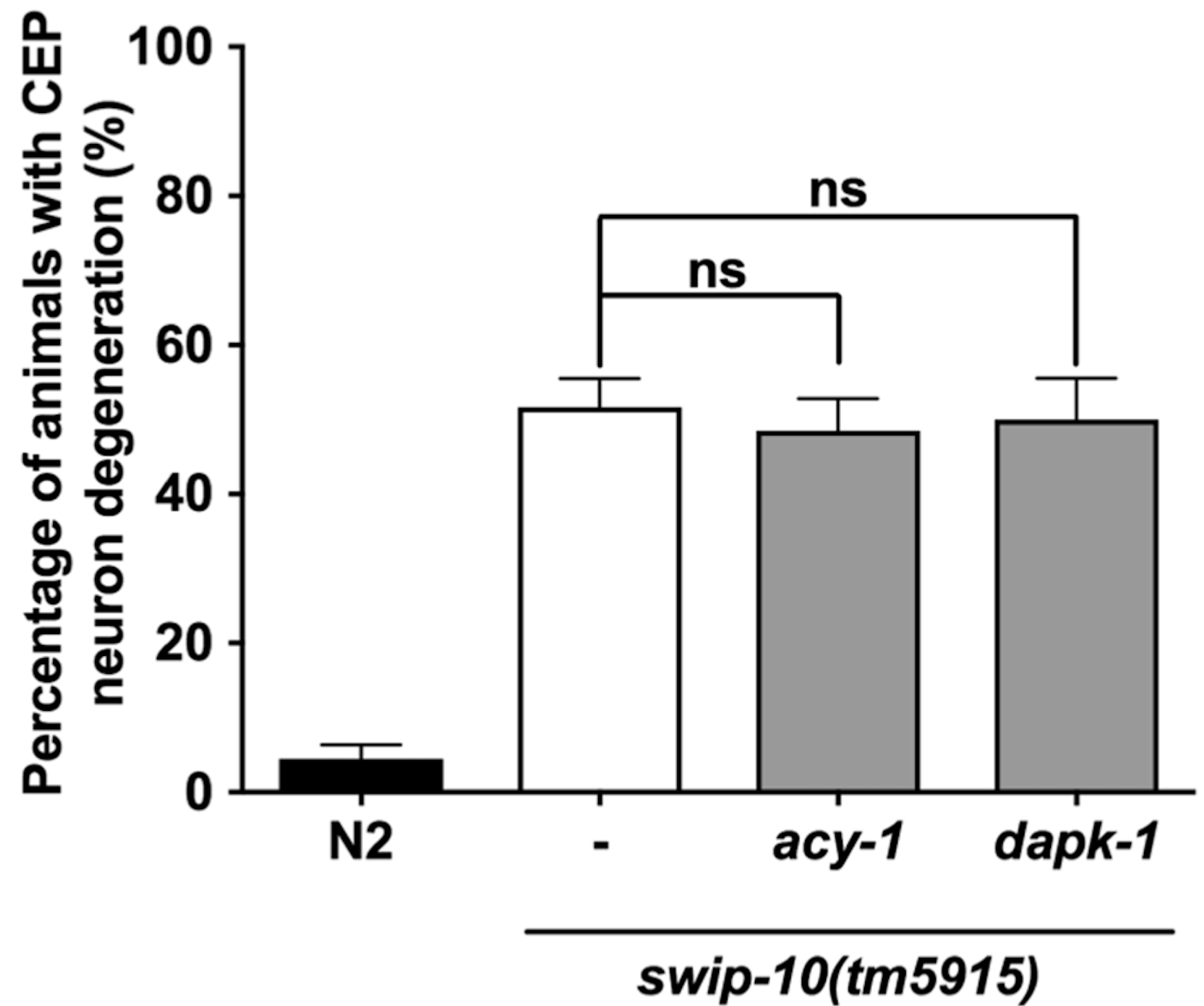

Supplement: S6 Fig — Data were analyzed by one-way ANOVA with Sidak’s post-tests, ns = non-significant (P>.05), error bars represent ± SEM, with n = 105–150 animals per strain. (TIF) [file pgen.1007269.s007.tif]
